# XX sex chromosome complement modulates immune responses to heat-killed *Streptococcus pneumoniae* immunization in a microbiome-dependent manner

**DOI:** 10.1186/s13293-024-00597-0

**Published:** 2024-03-14

**Authors:** Carly J. Amato-Menker, Quinn Hopen, Andrea Pettit, Jasleen Gandhi, Gangqing Hu, Rosana Schafer, Jennifer Franko

**Affiliations:** 1 Department of Microbiology, Immunology, and Cell Biology, West Virginia University School of Medicine, Morgantown, WV USA; 2 Department of Research, West Virginia University School of Dentistry, Morgantown, WV USA; 3 Present Address: National Institute of Neurological Disorders and Stroke, National Institute of Health, Bethesda, MD USA

**Keywords:** X chromosome, Four Core Genotype, HKSP, Gut microbiome, Kdm6a, SCFA, Sex differences, IgM, Plasma cells

## Abstract

**Background:**

Differences in male vs. female immune responses are well-documented and have significant clinical implications. While the immunomodulatory effects of sex hormones are well established, the contributions of sex chromosome complement (XX vs. XY) and gut microbiome diversity on immune sexual dimorphisms have only recently become appreciated. Here we investigate the individual and collaborative influences of sex chromosome complements and gut microbiota on humoral immune activation.

**Methods:**

Male and female Four Core Genotype (FCG) mice were immunized with heat-killed *Streptococcus pneumoniae* (HKSP). Humoral immune responses were assessed, and X-linked immune-related gene expression was evaluated to explain the identified XX-dependent phenotype. The functional role of *Kdm6a*, an X-linked epigenetic regulatory gene of interest, was evaluated ex vivo using mitogen stimulation of B cells*.* Additional influences of the gut microbiome on sex chromosome-dependent B cell activation was also evaluated by antibiotically depleting gut microbiota prior to HKSP immunization. Reconstitution of the depleted microbiome with short-chain fatty acid (SCFA)-producing bacteria tested the impact of SCFAs on XX-dependent immune activation.

**Results:**

XX mice exhibited higher HKSP-specific IgM-secreting B cells and plasma cell frequencies than XY mice, regardless of gonadal sex. Although *Kdm6a* was identified as an X-linked gene overexpressed in XX B cells, inhibition of its enzymatic activity did not affect mitogen-induced plasma cell differentiation or antibody production in a sex chromosome-dependent manner ex vivo. Enhanced humoral responses in XX vs. XY immunized FCG mice were eliminated after microbiome depletion, indicating that the microbiome contributes to the identified XX-dependent immune enhancement. Reconstituting microbiota-depleted mice with select SCFA-producing bacteria enhanced fecal SCFA concentrations and increased humoral responses in XX, but not XY, FCG mice. However, exposure to the SCFA propionate alone did not enhance mitogenic B cell stimulation in ex vivo studies.

**Conclusions:**

FCG mice have been used to assess sex hormone and sex chromosome complement influences on various sexually dimorphic traits. The current study indicates that the gut microbiome impacts humoral responses in an XX-dependent manner, suggesting that the collaborative influence of gut bacteria and other sex-specific factors should be considered when interpreting data aimed at delineating the mechanisms that promote sexual dimorphism.

**Supplementary Information:**

The online version contains supplementary material available at 10.1186/s13293-024-00597-0.

## Highlights

- Humoral immune responses against HKSP immunization are influenced by the possession of an XX vs. XY sex chromosome complement. While gonadal sex differentially influenced the number of antigen-specific IgM-secreting cells, the overall percentage of CD138 + plasma cells generated in response to HKSP immunization was not influenced by gonadal sex.
- *Kdm6a* is overexpressed in XX vs. XY B cells and splenocytes of HKSP-immunized mice and is demonstrated to be biallelically expressed in a subset of B cells.
- Ex vivo inhibition of KDM6a enzymatic activity promotes plasma cell differentiation in response to mitogenic stimulation. However, this effect was not sex chromosome-dependent. KDM6a inhibition did not impact total IgM concentrations in culture supernatants following mitogenic stimulation.
- XX-dependent immune enhancement is microbiome-dependent. Reconstitution of the antibiotic-depleted gut microbiome with select SCFA-producing bacteria rescued the XX-dependent immune phenotype observed in XX, but not XY, FCG mice.

**Supplementary Information:**

The online version contains supplementary material available at 10.1186/s13293-024-00597-0.

## Plain language summary

Male and female immune systems differ in their ability to respond to infectious challenge. While males tend to be more susceptible to infection and produce lower amounts of antibodies in response to vaccination, females are more prone to develop autoimmune and inflammatory diseases. Key contributors to these differences include sex hormones, sex chromosome complement (XX in females vs. XY in males), and distinct gut microbial communities capable of regulating immune activation. While each factor has been studied individually, this research underscores the potential for these factors to collaboratively impact immune activation. Here, possession of an XX vs. XY sex chromosome complement was demonstrated to enhance antibody responses to heat-killed *Streptococcus* *pneumoniae* vaccination. While attempting to determine the underlying cause of this immune enhancement, the gut microbiome was identified to play a critical role. In the absence of an intact gut microbiome, XX immune activation was reduced to levels similar to those seen in XY sex chromosome complement-possessing mice. Replacement of the depleted gut microbiomes with select SCFA-producing bacterial species enhanced SCFA levels in antibiotic-treated mice and rescued the XX-dependent immune enhancement, suggesting a SCFA-mediated contribution. Further studies are needed to determine exactly how these select bacteria impact immune activation in a sex chromosome complement-dependent manner. Our findings highlight the need to consider the collaborative effects of individual sex-specific factors when attempting to understand immune sex biases, as a better understanding of these interactions will likely pave the way for improving therapeutics and vaccines tailored to both sexes.

**Supplementary Information:**

The online version contains supplementary material available at 10.1186/s13293-024-00597-0.

## Background

Sex differences in immune responses have been well characterized [1]. In general, females elicit stronger humoral and cell-mediated responses to infection and respond better to vaccination than males, but in turn are more susceptible to autoimmune and inflammatory disorders [1–7]. Historically, hormonal differences have been described as the predominant determinant contributing to such sexual dimorphisms, as estradiol and progesterone are known to be immunostimulatory in nature and testosterone immunosuppressive [8–10]. However, the prevalence of immune sex differences before puberty and following the onset of menopause suggests a role for non-hormonal factors as well [1, 11–14].

Recently, the influence of the XX vs. XY sex chromosome complement on immune cell activation has become increasingly appreciated. The X chromosome encodes for a large number of immune-related genes, as well as epigenetic regulators associated with lymphocyte activation and differentiation [15]*.* While the dosages of X-linked genes are typically balanced between males and females via the process of X chromosome inactivation (XCI) [16], X-linked genes related to immunity have been demonstrated to more readily escape XCI in immune cells than other somatic cell types, and are uniquely regulated in B lymphocytes [17–21]. Multiple X-linked immune-related genes have been demonstrated to escape inactivation and their biallelic expression correlates with disease. For example, *CD40L and CXCR3* are biallelically expressed in T cells isolated from female, but not male, systemic lupus erythematosus (SLE) patients [22, 23]. *TLR7* and *TLR8* have also been identified as escape genes and are overexpressed in primary B lymphocytes, monocytes, and plasmacytoid dendritic cells isolated from female SLE patients and XXY Klinefelter syndrome males [20, 24]. In addition to SLE, sex chromosome complement-dependent influences have been identified in animal models of experimental autoimmune encephalitis (EAE) [25], anti-viral immunity [26], stroke-induced neuroinflammation [27, 28], and various metabolic disorders [29].

Sex has also been shown to influence the complexity and diversity of gut microbiome populations [30–33]. Using a panel of over 100 diverse inbred strains of mice, Org et al. identified distinct gut microbial communities in male vs. female mice whose composition was influenced by the presence of male vs. female sex hormones [31]. Reciprocally, distinct sex-specific gut microbial communities have also been demonstrated to modulate sex steroid production and influence immune cell activation by direct and indirect mechanisms. Differences in male vs. female susceptibility to type I diabetes have been directly linked to sex-specific gut microbiome populations capable of enhancing systemic androgen concentrations in male mice, or in female mice colonized with adoptively transferred male gut microbial communities [30, 32]. While these studies demonstrate that sex hormones and gut microbial communities collaborate to influence immune activation, to our knowledge no previous study has evaluated whether sex chromosome-dependent immune phenotypes are influenced by the gut microbiome.

Previously, our lab demonstrated that the immunomodulatory compound propanil (3,4-dichloropropionanalide) enhances immune responses to heat-killed *Streptococcus pneumoniae* (HKSP) immunization in an XX sex chromosome complement-dependent manner using Four Core Genotype (FCG) mice [34]. FCG mice exhibit one of four different genotypes: XX and XY females (ovaries) and XX and XY males (testes) and allow for the study of individual and collaborative sex hormone and sex chromosome complement-dependent effects [35–38]. While the mechanism mediating this propanil-mediated XX-dependent immune enhancement has yet to be defined, the contribution of circulating sex hormones was ruled out, as gonadectomy did not inhibit propanil-mediated immune enhancement [34]. Interestingly, in vivo, propanil is broken down into two major metabolites, 3,4-dichloroanaline (DCA) and the short-chain fatty acid (SCFA) propionate, via hepatic acylamidase-mediated hydrolysis [39]. Given that propionate, a SCFA with known immunomodulatory function, is also one of the major metabolic end-products of dietary fiber fermentation by gut microbiome bacteria [40–47], we hypothesized that the gut microbiome and/or its metabolic byproducts may collaboratively influence sex-specific immune outcomes in a sex chromosome-dependent manner.

In the following study, we evaluated the independent and collaborative influences of the XX vs. XY sex chromosome complements and sex-specific gut microbial communities on immune activation. Following immunization with HKSP, enhanced HKSP-specific antibody secreting cells and plasma cell differentiation were noted in XX vs. XY male and female FCG mice. While *Kdm6a*, an X-linked epigenetic regulator with known immunomodulatory function, was demonstrated to be overexpressed in XX-possessing cells and to be expressed from the inactive X chromosome in at least a subset of B cells, the ability of *Kdm6a* to influence plasma cell differentiation via its histone demethylase activity was not identified to be sex chromosome-dependent. This suggested that other XX-dependent regulatory factors must be contributing to the XX-dependent phenotype. Based on our previous work investigating propanil-mediated XX-dependent immune enhancement, we developed the novel hypothesis that endogenous SCFA-producing bacteria in the gut may influence immune activation in an XX-dependent manner. Antibiotic depletion of the endogenous microbiome reduced humoral responses to HKSP immunization in XX mice to levels similar to XY mice, but had no influence on XY responses. This demonstrates that stronger immune responses in XX animals were microbiome-dependent. Reconstitution of the antibiotically-depleted microbiota with specific SCFA-producing bacteria enhanced SCFA concentrations in the gut and restored the XX-dependent immune phenotype. Given that possession of an XX vs. XY sex chromosome complement had minimal impact on microbiota compositions, additional studies are warranted to evaluate the mechanism by which these SCFA-producing bacteria mediate their sex chromosome-dependent effect.

## Materials and methods

### Four Core Genotype model

B6.Cg-Tg(Sry)2Ei *Sry*^*dl1Rlb*^/ArnoJ (XY^−^*Sry*) male mice were originally purchased from The Jackson Laboratory (Bar Harbor, Maine). A colony was subsequently maintained at West Virginia University by breeding B6.Cg-Tg(Sry)2Ei *Sry*^*dl1Rlb*^/ArnoJ (XY^−^*Sry*) males with C57BL/6 J females (The Jackson Laboratory). All purchased animals were allowed to acclimate for one week prior to use. The sex determining region of the Y chromosome, the *Sry* gene, had previously been deleted from the Y chromosome of XY^−^*Sry* mice and inserted as a transgene onto autosome 3. Breeding of XY^−^*Sry* male mice with wildtype C57BL/6 female mice produced FCG mice: XX or XY gonadal females (XXF and XYF) and XX or XY gonadal males (XXM and XYM), as shown in Additional file 1: Figure S1. FCG mice were weaned at 21 days of age. The genotype of the offspring was determined by PCR amplification of the following genes: *Sry, Ymt*, and *Myo*, using DNA isolated from tail samples or ear punches obtained at weaning. QIAGEN Fast Cycling PCR kit (Qiagen, Louisville, KY) was used for PCR amplification.

Mice were housed in microisolator cages in specific pathogen-free conditions on a 12 h light–dark cycle with food and water provided *ad libitum*. Studies were conducted in accordance with all federal and institutional guidelines for animal use and were approved by the WVU Institutional Animal Care and Use Committee, protocol #1603001079 (Table 1).

**Table 1:** *Sry*, *Ymt*, and *Myo* primer sequences (Invitrogen):

Primer target	Sequence
*Myo*	Forward: 5ʹ-TTA CGT CCA TCG TGG ACA GCA T-3ʹ
	Reverse: 3ʹ-TGG GCT GGG TGT TAG TCT TAT-5ʹ
*Sry*	Forward: 5ʹ-AGC CCT ACA GCC ACA TGA TA-3ʹ
	Reserve: 3ʹ- TTG CCT GTA TGT GAT GG-5ʹ
*Ymt*	Forward: 5ʹ- GAG CTC TAC AGT GAT GA-3ʹ
	Reverse: 3ʹ-CAG TTA CCA ATC AAC ACA TCA C-5ʹ

### Preparation of heat-killed *Streptococcus pneumoniae* (HKSP) and immunization

*S. pneumoniae* strain R36A, an avirulent, nonencapsulated strain, was grown to mid-log phase in Todd-Hewitt broth + 1% yeast extract (Becton Dickinson, Sparks, MD) and stored at − 80 °C. For immunization, stock was cultured in a candle jar for 18 h at 37 °C on blood agar plates (Becton Dickinson). Colonies were selected and suspended in 200 ml broth, grown at 37 °C to an absorbance reading at 600 nm of 0.4 and heat-killed for 1 h in a 60 °C water bath. A final concentration of 10^9^ CFU/mL was established in PBS based on colony counts. Sterility was confirmed by culture and heat-killed *S. pneumoniae* stored at − 20 °C. Mice were immunized intraperitoneally with 2 × 10^8^ CFU HKSP, which elicits an optimal PC-specific antibody response 7 days post-vaccination [48, 49].

### Collection of samples for immunologic assessments

Mice were euthanized with 100 μl Euthasol (50 mg/ml, Virbac Inc., Fort Worth, TX) 7 days following immunization. Serum was collected by cardiac puncture. To generate single cell splenocyte suspensions, spleens were dissociated through 70 µM cell strainers (Thermo Fisher, Florence, KY) in RPMI-1640 (Corning, Manassas, VA), 10% heat inactivated fetal bovine serum (FBS, Hyclone Laboratories, Inc, Logan, UT), 10 mM HEPES (Sigma-Aldrich, Burlington, MA), 1 mM L-glutamine (Gibco, Rockville, MD), 5 × 10^−5^ M 2-mercaptoethanol (Sigma-Aldrich), 100 U/ml penicillin (Gibco), and 100 μg/ml streptomycin (Gibco). Red blood cells were lysed with Tris-buffered ammonium chloride. Cell suspensions were washed and counted using a hemacytometer. Viability was determined using Trypan blue dye exclusion (Sigma-Aldrich). When indicated, B cells were isolated from splenocytes by negative selection with the EasySep™ mouse B cell isolation kit (STEMCELL Technologies, Kent, WA). RNA was isolated from splenocytes or isolated B cells via Trizol:chloroform extraction or by the RNeasy Protect Mini Kit (Qiagen, Valencia, CA). Contaminating genomic DNA was eliminated using the Invitrogen TURBO DNA*-free*™ Kit (Thermo Fisher).

### Measurement of antibody-secreting cells (ASCs)

Millipore MultiScreen^®^ 96-well filter plates (Sigma-Aldrich) were coated with 50 μl phosphorylcholine (PC)-BSA (Biosearch Technologies, Petaluma, CA; 10 μg/ml) overnight at 4 °C. In subsequent steps, plates were washed with PBS + 0.01% Tween-20. Plates were blocked with 200 μl/well RPMI medium + 25% FBS for 2 h at 37 °C. Plates were washed and splenocytes (100 μl/well) added at 5 × 10^6^ cells/ml and 1 × 10^6^ cells/ml, each plated in triplicate. Plates were incubated for 4–6 h at 37 °C 5% CO_2_. After washing, goat anti-mouse alkaline phosphatase (AP) conjugated IgM antibody (Southern Biotechnology Associates, Birmingham, AL), diluted 1/2000 in PBS + 1% BSA + 0.05% Tween-20, were added to the appropriate wells (100 μl/well). Plates were incubated overnight at 4 °C and washed. Phosphatase substrate tablets (Sigma-Aldrich) were dissolved in water and 100 μl added to each well. Color development was stopped by washing with water. The number of spots/well was counted using a dissection microscope (ZEISS, Dublin, CA). The number of ASC was calculated using the mean number of spots from triplicate wells. Mice demonstrating an average of less than 20 spots in the 5 × 10^6^ dilution were considered non-responders and not included in subsequent analyses. The number of ASC was normalized to 1 × 10^6^ splenic B220 + B cells as determined by flow cytometric analysis.

### Flow cytometry

The Fc receptor of 200,000 cells were blocked with ChromPure IgG (Jackson ImmunoResearch, West Grove, PA) for 20 min, washed, and then stained with the following antibodies for 25 min on ice in the dark: rat anti-mouse B220-APC (RA3-6B2; BD Biosciences, San Diego, CA) and CD138-BV786 (281-2; BD). After staining, cells were washed and fixed in 0.04% paraformaldehyde (Thermo Fisher). Live cells were determined utilizing a Live/Dead Fixable Yellow Dead Cell Stain Kit (Invitrogen, Carlsbad, CA), and where applicable absolute cell number was determined using AccuCount beads (Spherotech, Lake Forest, IL). For each sample, 10,000–30,000 cells were collected for analysis (FCS Express software) on an LSRFortessa (BD).

### Gonadectomy surgeries

Bilateral castration or ovariectomy was performed on eight- to twelve-week-old mice by standard procedure [50]. Briefly, mice were anesthetized with isoflurane. Incisions were made through the skin and the underlying abdominal wall. The testes or ovaries were isolated and heated forceps used to cauterize the vas deferens and the blood vessel or transect the tip of the uterine horn and cauterize the blood vessels. The abdominal wall was closed with a suture and skin incisions closed with wound clips. Sham-operated mice (Sham) underwent the same procedure, but the testes or ovaries were left intact. Gonadectomized (Gdx) and Sham mice were housed four to five weeks following surgery before being used in experiments.

### Measurement of antibody concentrations by ELISA

Immulon 2 plates (ThermoLabsystems, Pittsburgh, PA) were coated overnight at 4 °C with goat anti-mouse human adsorbed unlabeled IgM (Southern Biotech; 100 μl/well). Plates were washed, blocked with 3% BSA in PBS at 37 °C overnight, washed, and 100 μl/well of four two-fold dilutions of sera in PBS + 1% BSA were added starting at 1:2. Sample containing plates were incubated for 1 h at 37 °C and washed. Goat anti-mouse AP conjugated antibodies (Southern Biotech; 100 μl/well) were added for 1 h at 37 °C. Plates were washed and 100 µl of phosphatase substrate tablets (Sigma-Aldrich) dissolved in p-Nitrophenyl Phosphate, Disodium Salt (PNPP) buffer was added to wells. Absorbance was read at 405 nm on an xMark™ Microplate Spectrophotometer with the Microplate Manager™ Software (Bio-Rad, Hercules, CA). Standard curves were generated using serial dilutions of purified rat anti-mouse IgM (Clone II/41, BD), and the 4-parameter fit equation used to calculate sample concentrations.

### RNA sequencing and analysis

After quantification and quality assessment of splenocyte RNA, 500 ng of total RNA was used to prepare Illumina-compatible libraries using the KAPA stranded mRNA library kit (Kapa Biosystems, Wilmington, MA). Sequencing was performed as 2 × 51 cycles on an Illumina HiSeq 2000 (Marshall Genomics Core). RNA-Seq data analysis followed previously described procedures [51, 52]. Briefly, RNA-Seq short reads were aligned to the mm10 with subread [53]. Read counts against RNA-Seq gene annotation was summarized with FeatureCounts [54]. Differentially expressed genes were predicted by EdgeR with FDR less than 0.1 and a log_2_FC more than 0.585. Gene expression values (RPKM; log_2_) across groups were visualized with GraphPad Prism version 9 for Windows (La Jolla, CA, www.graphpad.com).

### qRT-PCR

RNA concentrations and purity were measured on a Nanodrop 2000 (Thermo Fisher). cDNA was synthesized with the GoScript™ Reverse Transcription kit (Promega, Madison, WI). Transcripts were amplified by qRT-PCR using the primers below (Life Technologies, Carlsbad, CA) and the incorporation of PowerUp™ SYBR™ Green Master Mix (Life Technologies) was measured on the StepOnePlus RT-PCR system (Applied Biosystems, Foster City, CA) to determine expression levels. The following cycling conditions were utilized: 95 °C for 2 min, 40 cycles of (95 °C for 15 s—60 °C for 1 min), 95 °C for 15 s. *Kdm6a* and *Xist* expression were determined by normalization to *Gapdh* expression (Table 2).

**Table 2:** Primer sequences for qRT-PCR

Primer target	Sequence
*Gapdh*	Forward: 5ʹ-TTC ACC ACC ATG GAG AAG GC-3ʹ
	Reverse: 3ʹ-GGC ATG GAC TGT GGT CAT GA-5ʹ
*Kdm6a*	Forward: 5ʹ-TTC CTC GGA AGG TGC TAT TCA-3ʹ
	Reverse: 3ʹ-GAG GCT GGT TGC AGG ATT CA-5ʹ
*Xist*	Forward: 5ʹ-CAGAGTAGCGAGGACTTGAAGAG-3ʹ
	Reverse: 3ʹ-GCTGGTTCGTCTATCTTGTGGG-5ʹ

### Western blots

Total protein was isolated from HKSP-immunized mouse splenocytes using m-PER™ Mammalian Protein Extraction Reagent (Thermo Fisher) with 1% protease inhibitor (Cell Signaling, Danvers, MA). Protein concentrations were quantified using the Pierce™ Coomassie Plus (Bradford) Assay Kit (Thermo Fisher). Equal amounts of protein were boiled for 5 min and then resolved by SDS-Page on pre-cast Bolt 4–12% Bis–Tris Plus gels at 100 V for 60–120 min. The gel was electrophoretically transferred to polyvinylidene fluoride membranes using the iBlot™ 2 Transfer system (Invitrogen). Following transfer, membranes were blocked with 5% milk for one hour, followed by staining with primary antibody (anti-kdm6a 1:1000 and anti-β-tubulin 1:1000, Abcam, diluted in 5% milk) and incubated overnight. Membranes were then washed, incubated with secondary antibody (HRP anti-rabbit IgG) for one hour, then visualized using the SuperSignal West Pico PLUS substrate (Thermo Fisher). Blots were imaged on an iBright CL1500 (Invitrogen) imaging system and quantified using ImageJ with normalization to β-tubulin.

### RNA-FISH

Biallelic expression of *Kdm6a* in B cells and CD138 + plasma cells was observed using the Stellaris® RNA FISH system (Biosearch Technologies). Briefly, B cells from HKSP-immunized mouse spleens were purified by negative selection with the EasySep™ mouse B cell isolation kit (STEMCELL Technologies). Plasma cells from HKSP-immunized mouse spleens were purified by positive selection via FACS Aria III (BD Biosciences) after staining with CD138-BV786. 5 × 10^6^ B cells were washed with PBS and resuspended in 1 mL fixation buffer. After 10 min at room temperature, cells were washed three times with 1X PBS and then permeabilized in 1 mL of 70% ethanol for at least one hour at 4 °C. Cells were washed and resuspended in hybridization buffer containing the *Xist* (mouse *Xist* with Quasar^®^ 570 Dye, Biosearch Technologies) and/or *Kdm6a* (custom from Biosearch Technologies with Quasar^®^ 670; sequences available upon request) probes and incubated at 37 °C overnight in the dark. Cells were washed thoroughly and resuspended in 30µL of ProLong™ Glass Antifade Mountant with NucBlue™ (Invitrogen) and 5-10 µl mounted on *Superfrost Plus* microscope slides (Thermo Fisher). 2D images and Z-stacks were acquired on an inverted Nikon TI-E microscope with an A1R dual Galvano/resonant scanning confocal system equipped with four lasers (405 nm, 488 nm, 561 nm, 640 nm) and analyzed with NIS-Elements Advanced Research. For each sample, five sections were imaged and the following quantified: total number of cells, number of cells with an *Xist* cloud, and number of cells with *Xist* and *Kdm6a* colocalization.

### Ex vivo splenocyte stimulation

Splenocytes isolated from naïve mice were cultured at 0.5 × 10^6^ cells/mL in 12-well tissue culture plates using the following culture media: RPMI-1640 (Corning), 10% heat inactivated fetal bovine serum (FBS, Hyclone Laboratories), 10 mM HEPES (Sigma-Aldrich), 1 mM l-glutamine (Gibco), 5 × 10^−5^ M 2-mercaptoethanol (Sigma-Aldrich), 100 U/ml penicillin (Gibco), and 100 μg/ml streptomycin (Gibco). Cells were stimulated using LPS (5 µl/mL, *E. coli* O55:B5; Sigma-Aldrich) and recombinant mouse IL-4 (0.1 µg/mL, R&D Systems, Minneapolis, MN). To study the effects of KDM6a inhibition, cells were incubated with 0.25, 0.5, or 2.0 µM GSK J4 or GSK J5 (R&D Systems) reconstituted in DMSO or with DMSO alone for 30 min prior to stimulation. To examine the impact of SCFA on plasma cell differentiation, cells were exposed to propionate dissolved in culture media (0.5, 1, and 2 mM; Sigma-Aldrich) 30 min prior to stimulation with LPS and IL-4. All cell cultures were supplemented with LPS and IL-4 (at half concentration of initial stimulation) every 24 h during the experiment.

### Microbiome assessment

Total DNA was extracted from fecal samples using the DNeasy PowerSoil DNA isolation kit (Qiagen) according to the manufacturer’s recommended protocol. PCR amplification of the V3 region of the 16s rRNA gene was performed by utilizing high pressure liquid chromatography-purified primers (Integrated DNA Technologies; Coralville, IA), AccuPrime PCR Kit (Invitrogen) and cycling conditions previously described by Fadrosh et al*.* [55]. Briefly, cycling conditions included: 95 °C for 6 min denature; 95 °C for 2 min, 50 °C for 2 min, 72 °C for 2 min 30 cycles; 72 °C for 4 min extend. Each reaction contained 0.5 μl Taq polymerase, 5 μl 10 × buffer 1(600 mM Tris-SO4 (pH 8.9), 180 mM (NH4)2SO4, 20 mM MgSO4, 2 mM dGTP, 2 mM dATP, 2 mM dTTP, 2 mM dCTP, thermostable AccuPrime™ protein, 10% glycerol), 20 μM forward primer, 20 μM reverse primer, and up to 60 ng DNA in a total volume of 50 μl. Primer sequences are available upon request. Following quantitation and quality control analysis of the amplified 16s rRNA amplification product, paired-end sequencing (2 × 150 bp) was performed using the Illumina MiSeq located in the Genomics Core Facility at WVU.

Microbiome sequencing files were analyzed using QIIME2 (version 2020.11) [56, 57]. Sequencing quality was inspected using fastQC [58]. DADA2 [59] was used to optimize the parameter for quality control and read trimming. Taxonomy assignments were performed using the SILVA 132 [60] database at 97% identities. Rarefaction curves of alpha diversity was used to estimate the sampling completeness for OTU and Shannon Diversity Index calculations. Beta diversity metrics calculated without rarefaction included Jaccard distances, unweighted UniFrac distances, weighted UniFrac distances, and Bray–Curtis distances. Significance in the difference between alpha and beta diversities was based on Kruskal–Wallis and permutational multivariate analysis of variance (PERMANOVA), respectively, and PCoA plots were generated in QIIME2.

### Short-chain fatty acid concentrations

Fecal samples were collected on dry-ice and stored at 80 °C until analysis. Concentrations of eight short-chain fatty acids: acetic acid (C2, acetate), propionic acid (C3, propionate), isobutyric acid (C4), butyric acid (C4, butyrate), 2-methyl-butyric acid (C5), isovaleric acid (C5), valeric acid (C5) and caproic acid (hexanoic acid, C6) were assessed by LC–MS/MS (Metabolon, Morrisville, NC), using their Metabolon Method TAM135: “LC–MS/MS Method for the Quantitation of Short Chain Fatty Acid (C2 to C6) in Human Feces” workflow.

### Microbiome depletion

Endogenous gut microbiomes were depleted using an antibiotic cocktail containing metronidazole (10 mg/ml, Sigma-Aldrich), vancomycin (10 mg/ml, Sigma-Aldrich), neomycin (20 mg/ml, Sigma-Aldrich) and ampicillin (20 mg/ml, VWR, Radnor, PA) in sterile water. The antibiotic cocktail (100 µl) was administered via oral gavage every day for 3 days and then every other day until the end of the experiment. Controls received sterile water alone. Chow was removed from all cages 4 h prior to antibiotic or water gavage to optimize antibiotic absorption. Microbiome depletion was verified using the LIVE/DEAD^®^ BacLight™ Bacterial Viability and Counting Kit (Life Technologies) and subsequent acquisition on the LSRFortessa flow cytometer (BD). For all experiments using antibiotics, mice were housed by group to eliminate cross-contamination from fecal ingestion, provided with autoclaved drinking water and irradiated chow throughout the experiment, and provided with clean autoclaved cages after each antibiotic treatment.

### Culture of SCFA-producing bacteria

The bacterial strains *Bifidobacterium longum*, *Clostridium symbiosum*, and *Lactobacillus fermentum* were purchased from ATCC. All strains were cultured in brain heart infusion (BHI) medium (Sigma-Aldrich). *L. fermentum* was cultured in aerobic conditions, while *B. longum and C. symbiosum* were cultured anaerobically using anaerobic gas jars, EZ gas packs (BD), and pre-reduced media. Under sterile conditions, bacteria were inoculated in 5-6 mL culture medium and incubated at 37 °C for two days. Secondary inoculations were done inoculating 1 mL of the initial culture into 5-6 mL of fresh culture medium. Cultures were then allowed to incubate at 37 °C for 1–2 additional days. Once OD values reached at least 0.8, as measured on the xMark™ Microplate spectrophotometer (Bio-Rad), serial dilutions were made and their ODs measured. Dilutions were then plated on pre-reduced Brucella Agar with 5% sheep blood plates (*B. longum* and *C. symbiosum*; Anaerobe Systems, Morgan Hill, CA) or Blood Agar (*L. fermentum;* TSA with sheep blood, Remel, Lenexa, KS) plates and incubated overnight at 37 °C. CFUs were counted and growth curves generated to establish standard curves for each bacterial species. Bacteria were then frozen in pre-reduced glycerol and stored at -80 °C for future use. Fresh cultures were initiated from frozen stocks 6 days prior to the first day they were needed in an experiment, with new inoculations into fresh media every other day. DNA was isolated from individual bacterial colonies and amplified by PCR using 16s primers (Eurofins Genomics, Louisville, KY). Sequencing of amplified PCR products allowed for comparison of sequences to known BLAST database for species confirmation. 16s primer sequences were as follows: Forward: 5ʹ-CGG TTA CCT TGT TAC GAC TT-3ʹ. Reverse: 5ʹ-AGA GTT TGA TCC TGG CTC AG-3ʹ.

### Reconstitution of SCFA-producing bacteria and inulin administration

The endogenous microbiome was depleted in all mice by antibiotic gavage as described above, with mice receiving antibiotics daily for 3 days. To reconstitute the microbiome with SCFA-producing bacteria, mice received a cocktail containing the following bacteria: *B. longum* (1 × 10^7^), *C. symbiosum* (5 × 10^6^), and *L. fermentum* (1 × 10^9^) via oral gavage on Days 4 and 5. Bacterial counts were determined by OD measurements at 600 nm and previously established standard growth curves. Control mice received oral gavage of sterile medium alone. Inulin (MilliporeSigma) was diluted in sterile water and provided as a second oral gavage (10 mg in 100 µl) on Days 4 and 5. Mice not receiving inulin were provided a second oral gavage of sterile water (100 µl) alone. All mice were then immunized (i.p.) with 2 × 10^8^ CFU HKSP on day 6. Mice receiving inulin alone continued to receive antibiotics in sterile water gavage every other day throughout the experiment, while the experimental groups received oral gavage of sterile water alone. Fecal pellets were collected at Day 0 (pre-antibiotics), Day 4 (post-antibiotics and pre-bacteria ± inulin gavage), Day 6 (post-bacteria ± inulin, pre-HKSP immunization), and Day 13 (euthanasia) and assessed for gut colonization status by the LIVE/DEAD^®^ BacLight™ Bacterial Viability and Counting Kit (Life Technologies). SCFA levels were assessed by LC–MS/MS (Metabolon, Morrisville, NC).

### Statistics

**Fig. 1:**
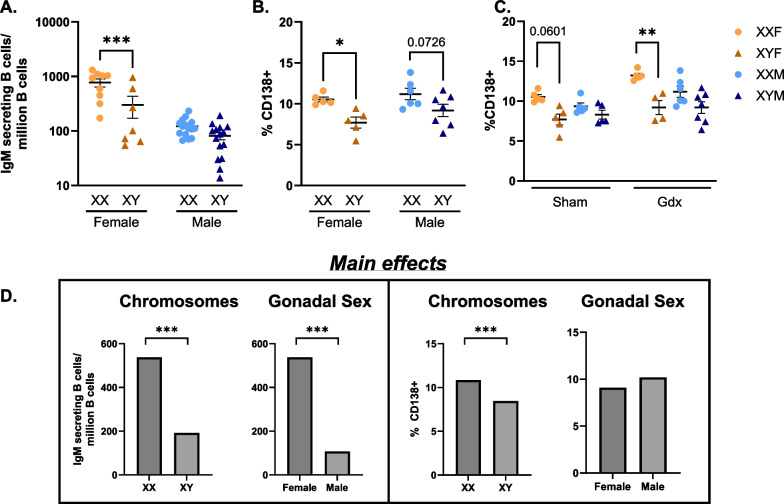
Possession of an XX vs. XY sex chromosome complement influences humoral responses to HKSP immunization. Immune responses against HKSP were assessed in FCG females and males one-week post-HKSP immunization. Numbers of HKSP-specific IgM-secreting B cells (**A**) and percentages of CD138 + plasma cells (**B**) were measured using ELISpot and flow cytometry, respectively. To assess the impact of sex hormones on sex chromosome-dependent phenotypes, percentages of CD138 + plasma cells were assessed in sham-operated (Sham) vs. gonadectomized (Gdx) female and male FCG mice following the same immunization protocol (**C**). Main effects of chromosomes and gonadal sex on variables assessed in A and B are presented by graphing the predicted (LS) means of each variable (**D**). Data in A-C are represented as the mean ± SEM with each data point representing one mouse. Statistical analyses by two-way ANOVA followed by Sidak’s multiple comparisons tests (**A, B**) or three-way ANOVA followed by Tukey’s multiple comparisons tests (**C**). Comparisons in graphs are representative of multiple comparisons tests; *p < 0.05; **p < 0.01; ***p < 0.001. ANOVA main and interactive effects are provided in Additional file 9: Tables S1-S2

Statistical analyses were performed in GraphPad Prism (San Diego, CA). Data are represented as the mean ± SEM with each data point representing one mouse and statistical significance set as *p* < 0.05 unless otherwise indicated. Two-way ANOVA tests were utilized to evaluate main and interactive effects in the FCG mouse model as previously established [37, 61, 62], with factors of sex chromosome complement (XX vs. XY) and gonadal sex (female vs. male). Three-way ANOVA tests were utilized in data sets with a third independent variable (treatment or sham-operated vs. gonadectomized) to assess for main and interactive effects. ANOVAs were followed by Sidak’s or Tukey’s multiple comparisons test as indicated. Statistical tests utilized are denoted in the figure legends. Main effect graphs in Fig. 1 were generated by plotting the predicted (LS) means of XX vs. XY and female vs. male based on the accompanying two-way ANOVA analyses. Statistical analyses of the gut microbiome bacteria alpha and beta diversities were based on Kruskal–Wallis and permutational multivariate analysis of variance, respectively.

## Results

### The sex chromosome complement influences humoral responses to HKSP immunization

To evaluate whether XX vs. XY sex chromosome complements differentially regulate sexually dimorphic humoral immune responses to HKSP immunization, the FCG mouse model was utilized (Additional file 1: Fig. S1; [35, 36, 63]). Ovary-bearing females with XX (XXF) or XY (XYF) sex chromosomes and testes-bearing males with XX (XXM) or XY (XYM) sex chromosomes were immunized with HKSP. One-week post-immunization, the numbers of HKSP-specific IgM-antibody secreting cells (ASC) were assessed (Fig. 1A). Consistent with previously published reports demonstrating enhanced immune responses against *Streptococcus pneumoniae* in females vs. males [64–66], two-way ANOVA identified a main effect of gonadal sex (females > males; *p* < 0.0001), with FCG females producing significantly higher numbers of HKSP-specific IgM ASC than males. An additional main effect of chromosome complement (XX > XY; *p* = 0.0006) was also identified, indicating stronger responses in XX vs. XY animals, regardless of gonadal sex (Additional file 9: Table S1). A two-way interaction (*p* = 0.0032; Additional file 9: Table S1) between gonadal sex and sex chromosomes was identified, demonstrating a potential interactive effect of gonadal sex and sex chromosome complement on the number of HKSP-specific IgM ASC generated in response to immunization. Due to the magnitude of the immune response in females vs. males, the XX-dependent phenotype in males may be masked by the main effect of gonadal sex. If the influence of the XX vs. XY sex chromosome complement is evaluated separately in male mice only (unpaired t-test), a similar XX-dependent effect on HKSP-specific IgM ASC is identified (*p* = 0.0272).

XXF female mice also exhibited increased percentages of CD138 + plasma cells when compared with XYF female mice (*p* = 0.0227; Fig. 1B). Despite not reaching statistical significance, CD138 + plasma cell frequencies trended similarly in XXM vs. XYM males (*p* = 0.0726; Fig. 1B). Using two-way ANOVA analyses, only the main effect of the sex chromosomes was significant (XX > XY; *p* = 0.0020; Additional file 9: Table S1) with XX responses greater than XY. Unlike the main effect of gonadal sex in the numbers of ASC, gonadal sex did not influence the frequency of CD138 + plasma cells (*p* = 0.1258; Additional file 9: Table S1), emphasizing the role of the sex chromosome complement in humoral responses independently of gonadal sex.

To confirm that circulating gonadal hormones were not impacting plasma cell frequencies, percentages of CD138 + plasma cells were assessed in gonadectomized or sham-operated FCG mice. While no gonadal sex main effect was identified (*p* = 0.1508), the main effect of the sex chromosomes was enhanced (*p* < 0.0001). Similar to the antigen-specific ASC responses, plasma cell frequencies were higher in XX vs. XY females, with XX vs. XY males trending in the same fashion (Fig. 1C, Additional file 9: Table S2).

### Identification of *Kdm6a* as an X-linked gene that is overexpressed in XX B cells

**Fig. 2:**
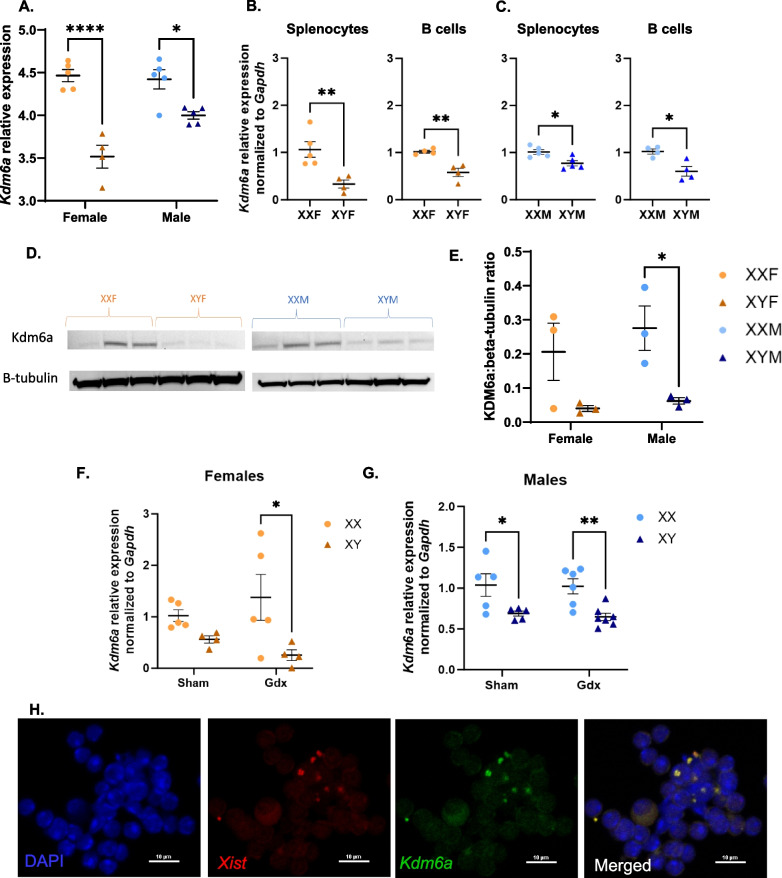
*Kdm6a* is differentially expressed in XX vs. XY splenocytes and B cells isolated from HKSP-immunized FCG mice. One-week post-HKSP immunization, RNA-Sequencing was performed on splenocytes isolated from male and female FCG mice. Levels of *Kdm6a* expression are represented as relative expression using EdgeR values (**A**). Differential expression of *Kdm6a* was confirmed by qRT-PCR using both splenocytes and B cells isolated from HKSP-immunized females (**B**) and males (**C**). KDM6a protein levels were assessed by western blot using lysates generated from HKSP-immunized FCG splenocytes (**D, E**). Relative levels of protein expression were quantified as KDM6a:beta-tubulin ratios (**E**). *Kdm6a* expression was also assessed in B cells from HKSP-immunized gonadectomized (Gdx) and sham-operated (Sham) females (**F**) and males (**G**, Additional file 9: Table S4). RNA-FISH was utilized to determine if *Kdm6a* was expressed from the inactivate X chromosome in B cells isolated from female XX FCG mice previously immunized with HKSP. Representative images of individual DAPI, *Xist*, and *Kdm6a*, as well as merged images, are presented (**H**). Colocalization of *Kdm6a* and *Xist* signals was considered indicative *Kdm6a* expression from the inactive X chromosome. Data are represented as the mean ± SEM with each data point representing one mouse. Statistical analyses by two-way ANOVA followed by Sidak’s multiple comparisons test (**A, E**), unpaired t-tests (**B, C**), or by three-way ANOVA followed by Tukey’s multiple comparisons test (**F**, **G**). Comparisons in graphs are representative of multiple comparisons tests; *p < 0.05; **p < 0.01; ****p < 0.0001. ANOVA main and interactive effects for **A** & **E** are provided in Additional file 9: Table S3

Given that HKSP-specific immune responses were enhanced in XX vs. XY mice, we hypothesized that X-linked gene dosage effects may be important. To test this hypothesis, RNA-Seq was performed on splenocytes isolated from HKSP-immunized male and female FCG mice. As anticipated, *Xist* and *Sry* were identified as genes overexpressed in XX vs. XY cells and male vs. female cells, respectively (Additional file 3: Fig. S3 and Additional file 9: Table S5). Only two additional X-linked genes were demonstrated to be overexpressed in XX vs. XY splenocytes isolated from male and female FCG mice immunized with HKSP (threshold of log2FC > 0.585 and FDR < 0.1): *Eif2s3x*, and *Kdm6a*. KDM6a (Lysine (K)-specific demethylase 6A, aka UTX) is a histone demethylase whose epigenetic regulatory function has previously been demonstrated to modulate other immune cells in an XX-dependent manner [67, 68], making it an interesting candidate gene for our studies. The increased expression of *Kdm6a* in XX vs. XY splenocytes (Fig. 2A, Additional file 9: Table S3; XXF vs. XYF females *p* < 0.0001; XXM vs. XYM males *p* = 0.0214) was confirmed by qRT-PCR in both total splenocytes and B cells isolated from HKSP-immunized females (Fig. 2B) and males (Fig. 2C). Protein concentrations were also demonstrated to be significantly higher in B cells isolated from XXM vs. XYM males (*p* = 0.0439) and trended in a similar fashion for XXF vs. XYF females (Fig. 2D, E) as determined by western blot.

The differential expression of *Kdm6a* was also evaluated in B cells isolated from XX vs. XY sham-operated and gonadectomized female (Fig. 2F) and male (Fig. 2G) mice immunized with HKSP. Similar trends for *Kdm6a* overexpression were noted in XX vs. XY mice regardless of gonadectomy or intact gonads (Additional file 9: Table S4, Main Effect Chromosomes (XX, XY)), suggesting that the XX vs. XY differential expression of *Kdm6a* is regulated independent of circulating sex hormones.

Increased expression of an X-linked gene could indicate either biallelic expression or increased expression from the active X chromosome. To investigate whether *Kdm6a* is expressed from the inactive X chromosome, RNA-FISH was performed on B cells isolated from XXF female FCG mice one-week post-HKSP immunization. The inactive X chromosome was detected using a fluorescent probe targeting *Xist*, a long, non-coding RNA that coats the inactive X chromosome resulting in its inactivation and formation of an *Xist* cloud [69, 70]. Colocalization of *Xist* and *Kdm6a*-specific probes were considered indicative of *Kdm6a* being expressed from the inactive X chromosome (Fig. 2H). Approximately 13% of total B cells presented with an *Xist* cloud (Additional file 4: Fig. S4C), suggesting that they were activated in response to HKSP immunization, as naïve B cells have been shown to lack an *Xist* cloud [17, 71].

Of the total B cells possessing *Xist* clouds, 78% exhibited colocalization of *Kdm6a* with *Xist* RNA (Additional file 4: Fig. S4A, D), suggesting that *Kdm6a* can be expressed from the inactive X chromosome and may therefore be escaping XCI in a subset of B cells. Plasma cells were then specifically isolated. Interestingly, although plasma cells presented with an *Xist* cloud more frequently (64%), only 13% of plasma cells with *Xist* clouds exhibited *Xist*/*Kdm6a* colocalization (Additional file 4: Fig. S4B-D).

### Ex vivo inhibition of KDM6a activity promotes plasma cell differentiation, but not in a sex chromosome-dependent manner

**Fig. 3:**
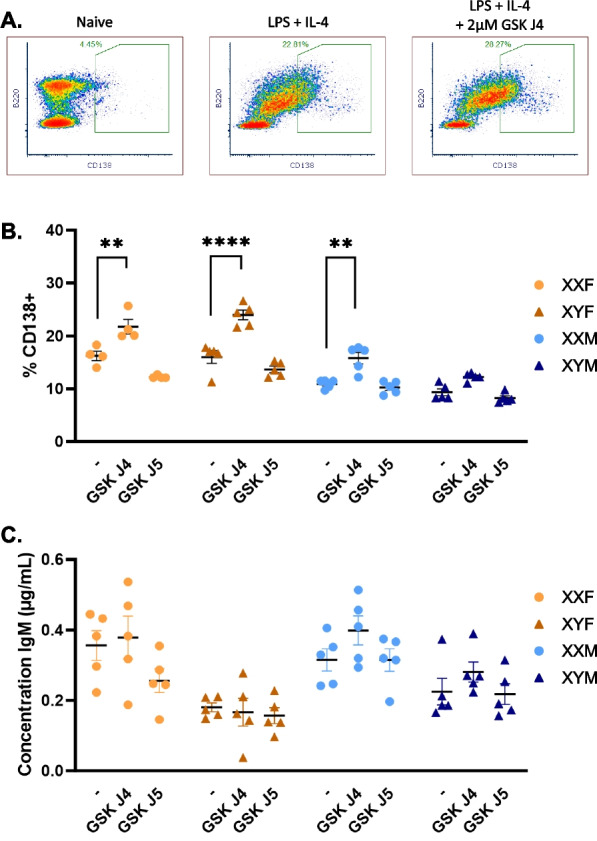
KDM6a inhibition enhances plasma cell differentiation, but not IgM secretion, similarly in all four genotypes. Splenocytes isolated from naïve FCG mice were stimulated ex vivo with IL-4 (0.01 µg/mL) and LPS (5 µg/mL) in the presence or absence of 2 µM GSK J4 or 2 µM GSK J5. Representative density plots of flow cytometric data from naïve (day 0) or stimulated splenocytes (day 3) ± GSK J4 exposure are depicted in (**A**). Percentages of CD138 + plasma cells in FCG mice were quantified by flow cytometry (**B**). Supernatants were collected and total IgM concentrations were assessed by ELISA for each stimulation condition (**C**). Data are represented as the mean ± SEM with each data point representing one mouse. Three-way ANOVA with Tukey’s multiple comparisons tests. Comparisons in graphs are representative of multiple comparisons tests; **p < 0.01; ****p < 0.0001. ANOVA main and interactive effects are provided in Additional file 9: Table S6. Results from additional concentrations of GSK J4 and GSK J5 are provided in Additional file 5: Fig. S5 and Additional file 9: Table S7

We next sought to determine whether inhibiting the enzymatic activity of KDM6a functions to modulate plasma cell differentiation in an XX-dependent manner. Splenocytes were isolated from naïve FCG mice and stimulated with IL-4 and LPS in the presence or absence of increasing concentrations of GSK J4 or its inactive isomer GSK J5. GSK J4 is a chemical inhibitor specific for H3K27me3 demethylases, including KDM6a [72, 73]. In all four genotypes (XXF, XYF, XXM, and XYM), GSK J4 (2 µM) enhanced CD138 + plasma cell frequencies following ex vivo stimulation. (Fig. 3B, Additional file 5: Fig. S5A). However, it did so similarly in all genotypes with no significant chromosomal main effect (Additional file 9: Table S6, Additional file 9: Table S7). Demonstrating the specificity of the GSK J4-mediated effect, its inactive isomer, GSK J5, did not impact plasma cell frequencies (Additional file 5: Fig. S5C). Additionally, total IgM concentrations were assessed in the supernatants of stimulated cells in the presence or absence of GSK J4. In contrast to plasma cell frequencies, ex vivo inhibition of KDM6a activity did not impact mitogen-induced IgM production (Fig. 3C, Additional file 5: Fig. S5B). Taken together, these data suggest that while KDM6a’s demethylase activity influences CD138 + plasma cell differentiation, it does not do so in a sex chromosome-dependent manner. Therefore, despite Kdm6a’s overexpression in XX vs. XY mice, its demethylase activity cannot explain the XX-specific humoral enhancement.

When comparing the in vivo (Fig. 1) vs. ex vivo (Fig. 3) studies, it is interesting to note the differing main effects of sex chromosomes on CD138 + frequencies and IgM production. The number of IgM ASC and IgM concentrations were influenced by chromosome complement in vivo (Fig. 1D) and ex vivo (Fig. 3C), respectively, with increased IgM responses noted in XX vs. XY mice. Enhanced plasma cell frequencies in XX vs. XY mice, however, were lost in ex vivo studies (Fig. 3B and Additional file 9: Table S6). This led us to question whether other extrinsic factors in the in vivo environment could be differentially regulating XX-dependent CD138 + plasma cell frequency enhancement if sex hormones were not (Fig. 1C).

### The gut microbiome is required for XX-specific immune enhancement

**Fig. 4:**
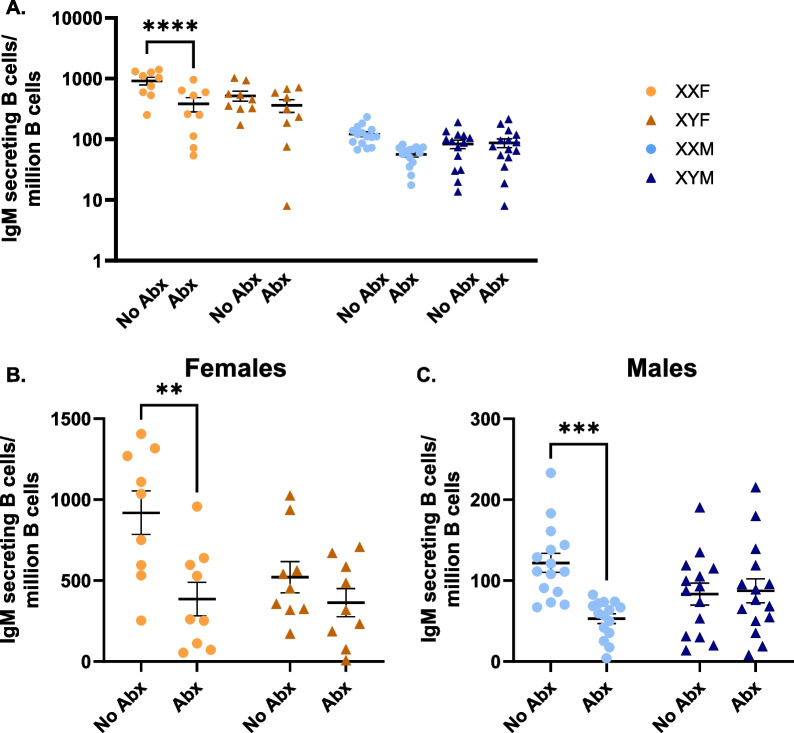
XX-specific enhancement in response to HKSP is dependent on the gut microbiome. The number of IgM-secreting B cells produced in response to HKSP immunization was assessed by ELISpot in male and female FCG mice possessing intact or antibiotically depleted gut microbiomes. Data are presented collectively for three-way ANOVA analyses (**A**) and separated by sex [females (**B**) and males (**C**)] for two-way ANOVA analyses to uncover chromosome roles independent of circulating sex hormones. Data are represented as the mean ± SEM with each point representing one mouse. Statistics by three-way ANOVA with Tukey’s multiple comparisons (**A**) or two-way ANOVA with Tukey’s multiple comparisons test (**B**, **C**). Significance indicated are representative of Tukey’s multiple comparisons tests. Comparisons in graphs are representative of multiple comparisons tests; **p < 0.01; ***p < 0.001; ****p < 0.0001. Abx = antibiotics. ANOVA main and interactive effects are provided in Additional file 9: Tables S8-S9

Sex biases in gut microbiome diversity have been reported and demonstrated to differentially influence immune activation [30, 74]. Here, we evaluated whether the gut microbiome could differentially influence immune activation in a sex chromosome complement-dependent manner. To deplete gut microbiota, FCG mice were administered an antibiotic (Abx) cocktail containing metronidazole (10 mg/ml), vancomycin (10 mg/ml), neomycin (20 mg/ml) and ampicillin (20 mg/ml) via oral gavage. Control animals received sterile water alone. On day 4, mice were immunized with HKSP. Similar to the experimental design of Fig. 1A, ELISpots were performed on day 10 to evaluate the number of HKSP-specific ASC (Additional file 2: Fig. S2; experimental design). Consistent with Fig. 1A, females produced more HKSP-specific IgM ASC than males in response to HKSP immunization regardless of antibiotic treatment (Fig. 4A, Additional file 9: Table S8: Main Effect—Gonadal Sex). Interestingly, antibiotic administration influenced HKSP-specific responses in a sex chromosome-dependent manner, significantly reducing XXF responses (*p* < 0.0001) to levels similar to those seen in XYF mice, but having no impact on XYF responses. Females (Fig. 4B) and males (Fig. 4C) were then analyzed separately (Additional file 9: Table S9), to account for any masking effects of the gonadal sex influence, and similar trends were observed in both females and males, where depletion of the gut microbiome in XX mice reduced IgM ASC, but had no impact on XY mice.

**Fig. 5:**
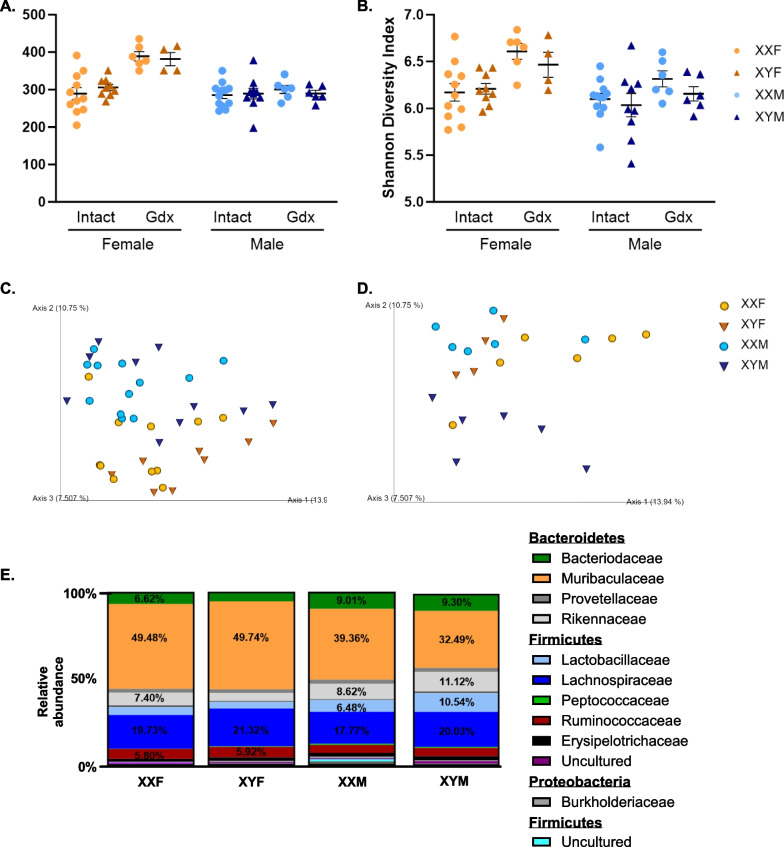
Assessment of gut microbiota diversity in FCG mice. 16s rRNA gene sequencing and metagenomic analyses were performed to assess microbiota diversity in FCG mice. The number of different OTUs as a function of the number of sequence reads (**A**) and Shannon Diversity Indexes (**B**) were determined for gonadally intact and gonadectomized (Gdx) animals. Representative PCoA plots of Bray–Curtis pair-wise comparison distances demonstrate clustering differences between males and females in both intact (**C**) and gonadectomized (**D**) animals. For each distinct OTU identified, percent abundancies were calculated for intact animals (**E**, Additional file 9: Table S12). The total height of y-axis represents 100% of the assigned sequences after quality filtering, and the size of the colored regions represents proportional contributions of each phylotype shown with the top 11 families being visualized. Abundances greater than 5% are labeled with percentages. Data in **A** and **B** are represented as the mean ± SEM with each point representing one mouse. Statistics by Kruskal–Wallis test for **A** and **B**, followed by Dunn’s multiple comparisons tests provided in Additional file 9: Table S10. Bray–Curtis comparisons between each group are provided in Additional file 9: Table S11. A full list of taxon abundancies in E are provided in Additional file 9: Table S12. Gdx = gonadectomized; OTUs = observable taxonomic units

Given the microbiome-dependent influence on XX vs. XY immune responses, the microbiota compositions of FCG mice were then characterized by 16s rRNA sequencing to determine if distinct microbiota in XX vs. XY mice could explain this phenotype. Based upon calculated alpha diversity metrics, namely Observed Taxonomic Units (OTUs) and Shannon diversity indexes, no significant differences were identified within XXF, XYF, XXM, and XYM samples collected from gonadally intact animals (Fig. 5A, B). Ovariectomy significantly enhanced alpha diversity in XX females (*p* = 0.0145, XX F Intact vs. Gdx), but did not influence alpha diversity in XY females, while castration had no influence on alpha diversity measured in males. Chromosome complement had no main effect in the alpha diversity in intact or gonadectomized animals (Additional file 9: Table S10).

Multiple beta diversity metrics were also calculated, including Bray–Curtis, Jaccard, unweighted UniFrac, and weighted UniFrac, to evaluate potential diversity between FCG groups. Representative Bray–Curtis principle coordinate analyses (PCoA) for intact (Fig. 5C) and gonadectomized (Fig. 5D) animals were generated. In addition, a summary of PERMANOVA Bray–Curtis distance comparisons, including gonadally intact and gonadectomized animals, can be found in Additional file 9: Table S11. Spatial segregation and clustering of male vs. female animals were noted and suggestive of compositional differences in their respective microbiota. Little influence of sex chromosome complement was apparent for male microbiota composition. While significant compositional differences were noted in XXF vs. XYF animals via Bray–Curtis, no differences were noted using Jaccard, unweighted UniFrac, or weighted UniFrac in gonadally intact animals (Table 3). This may indicate that the dissimilarity observed between XXF and XYF is largely due to changes in the abundance or presence of certain microbial species rather than differences in the overall composition or structure of the microbial communities. However, no significant differences were identified in the relative abundancies when comparing the top 11 most abundant bacterial families between XXF vs. XYF females. Figure 5E represents the relative abundance of taxa identified in FCG microbiomes and emphasizes the compositional similarities between XX vs. XY animals of the same gonadal phenotype. A full list of abundancies are provided in Additional file 9: Table S12.

**Table 3:** FCG microbiome diversity: statistical analysis of Beta-diversity indexes

Comparison	Jaccard	Unweighted UniFrac	Weighted UniFrac	Bray–Curtis
XXF vs. XYM	0.033*	0.121	0.032*	0.005*
XXF vs. XYF	0.111	0.555	0.112	0.034*
XXM vs. XYM	0.075	0.105	0.104	0.190
XXF vs. XXM	0.006*	0.096	0.258	0.003*
XYF vs. XYM	0.064	0.187	0.292	0.014*
XYF vs. XXM	0.001*	0.039*	0.064	0.001*

*p ≤ 0.05; comparison of index distances using QIIME2 plugins using PERMANOVA

**Fig. 6:**
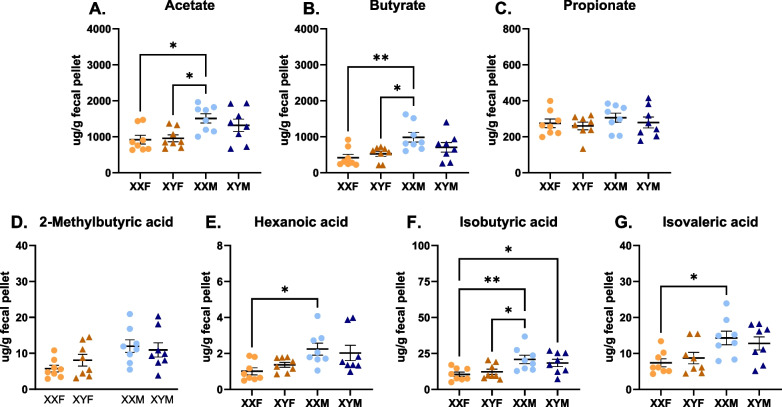
Concentrations of SCFA in the feces of male and female FCG mice. Fecal pellets from naïve FCG mice were collected and analyzed for a panel of short-chain fatty acids: acetate (**A**), butyrate (**B**), propionate (**C**), 2-methylbutyric acid (**D**), hexanoic acid (caproic acid, **E**), isobutyric acid (**F**), and isovaleric acid (**G**) by LC–MS/MS (Metabolon). Data are represented as the mean ± SEM with each point representing one mouse. Statistics by two-way ANOVA with Tukey’s multiple comparisons test. Comparisons in graphs are representative of multiple comparisons tests; *p < 0.05; **p < 0.01. ANOVA main and interactive effects are provided in Additional file 9: Table S13

Since compositional differences could not fully explain the microbiome-mediated enhancement of immune responses in XX mice, it was hypothesized that sex chromosome-dependent differences in concentrations of SCFAs, major metabolites of the gut microbiome with known immunomodulatory function, may contribute. To test this, fecal concentrations of seven distinct SCFAs, including acetate (C2), propionate (C3), and butyrate (C4), were measured using LC–MS/MS. While the concentration of individual SCFAs varied, in general, higher SCFA concentrations were measured in fecal pellets collected from male vs. female mice (Additional file 9: Table S13). Concentrations did not vary between XXF vs. XYF females and between XXM vs. XYM males FCG mice (Fig. 6).

**Fig. 7:**
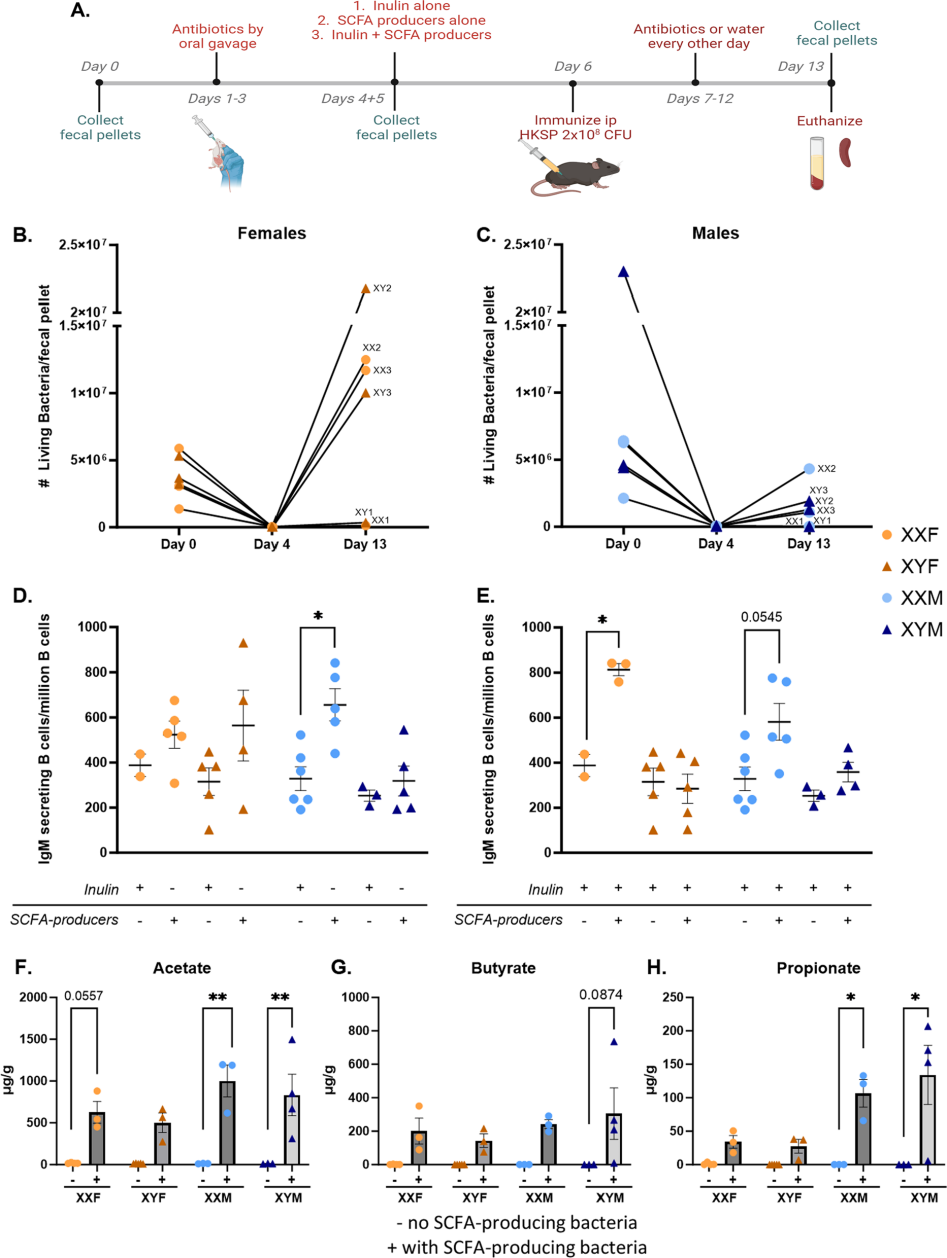
Reconstitution of gut microbiota with SCFA-producing bacteria increased humoral responses in an XX-dependent manner. Overview of experimental design (**A**). Briefly, gut microbiota of FCG mice were depleted using antibiotic oral gavage (3 days). On Day 4, mice were administered one of the following via oral gavage: inulin alone (Group 1); SCFA-producing bacteria alone (Group 2); or Inulin + SCFA-producing bacteria (Group 3). On Day 6, all mice were immunized with 2 × 10^8^ CFU heat-killed *Streptococcus pneumoniae*. Mice in the inulin alone exposed group received antibiotics by oral gavage every other day through Day 12, while mice receiving SCFA-producing bacteria ± inulin received water. Mice were euthanized on Day 13 and samples collected for immune response evaluations. Fecal pellets were collected on Day 0, Day 4 (prior to gavage treatments), and Day 13. The number of living bacteria per pellet for females (**B**) and males (**C**) was assessed by flow cytometry to assess successful gut microbiota depletion and reconstitution. Immune responses were evaluated as numbers of IgM-secreting B cells and analyzed as Inulin alone vs. SCFA-producing bacteria (**D**) or as Inulin alone vs. Inulin + SCFA-producing bacteria (**E**). Concentrations of acetate (**F**), butyrate (**G**), and propionate (**H**), three well-studied SCFAs with immunomodulatory function, were assessed by LC–MS/MS (Metabolon) to confirm effective SCFA-producing bacteria recolonization and subsequent SCFA production. The concentrations of additional SCFAs are provided in Additional file 7: Fig. S7. Data point labels in **B** and **C** indicate the sex chromosome complement (XX or XY) and the group number as indicated in **A**. Data are represented as the mean ± SEM with each point representing one mouse. Statistics by three-way ANOVA with Tukey’s multiple comparisons test. Comparisons in graphs are representative of multiple comparisons tests; *p < 0.05. ANOVA main and interactive effects are provided in Additional file 9: Tables S14 and S15

While the lack of differences in SCFA concentrations in the fecal pellets of XX vs. XY animals could have been suggestive that these metabolites were not contributing to XX-dependent immune enhancement, our laboratory previously demonstrated that the immunomodulatory compound propanil enhances responses to HKSP immunization in XX vs. XY animals when exposed to the same dose of the compound [34]. Given that propionate is a metabolite of propanil, we hypothesized that similar concentrations of SCFAs could impact immune activation differently in XX vs. XY animals. To evaluate this potential, the endogenous gut microbiomes of male and female FCG mice were depleted using antibiotics, as described earlier. Their endogenous microbiome was then reconstituted with select SCFA-producing bacteria in the presence or absence of inulin. Inulin is a fiber source that is metabolized into SCFAs, thereby enhancing SCFA concentrations when select SCFA-producing bacteria are present. Control groups underwent antibiotic microbiome depletion and inulin administration but received no SCFA-producing bacteria. All mice were then immunized with HKSP, and immune responses evaluated one week later (Fig. 7). Live/Dead flow staining of fecal content, as well as assessment of SCFA concentrations, demonstrated successful depletion of the endogenous gut microbiome and successful reconstitution in mice receiving SCFA-producers following initial antibiotic administration (Fig. 7B, C, F–H, Additional file 6: Fig. S6-7). When microbiomes were reconstituted with SCFA-producing bacteria only (Fig. 7D), XXM males exhibited a significant increase in HKSP-specific ASC when compared with XXM males that received inulin alone (*p* = 0.0479). When administered inulin and SCFA-producing bacteria (Fig. 7E), a similar trend was noted for XXM males (*p* = 0.0545). Similarly, XXF females receiving SCFA-producing bacteria and inulin generated significantly greater numbers of HKSP-specific ASC in response to HKSP immunization compared with XXF females that received inulin alone (*p* = 0.0234). Interestingly, SCFA-producing bacteria did not increase XY female or male immune responses above that of inulin controls, even though SCFA concentrations were confirmed to be increased in mice receiving SCFA-producing bacteria for all genotypes (Fig. 7F-H, Additional file 7: Fig. S7, Additional file 9: Table S15). Three-way ANOVA analyses (Additional file 9: Table S14) demonstrate a main effect of the sex chromosome complement (*p* < 0.0001) when SCFA-producing bacteria are provided with inulin, and a two-way interaction between the treatment and sex chromosomes (*p* = 0.0038), suggesting that gut-resident SCFA-producing bacteria influence immune responses differently in XX vs. XY animals.

**Fig. 8:**
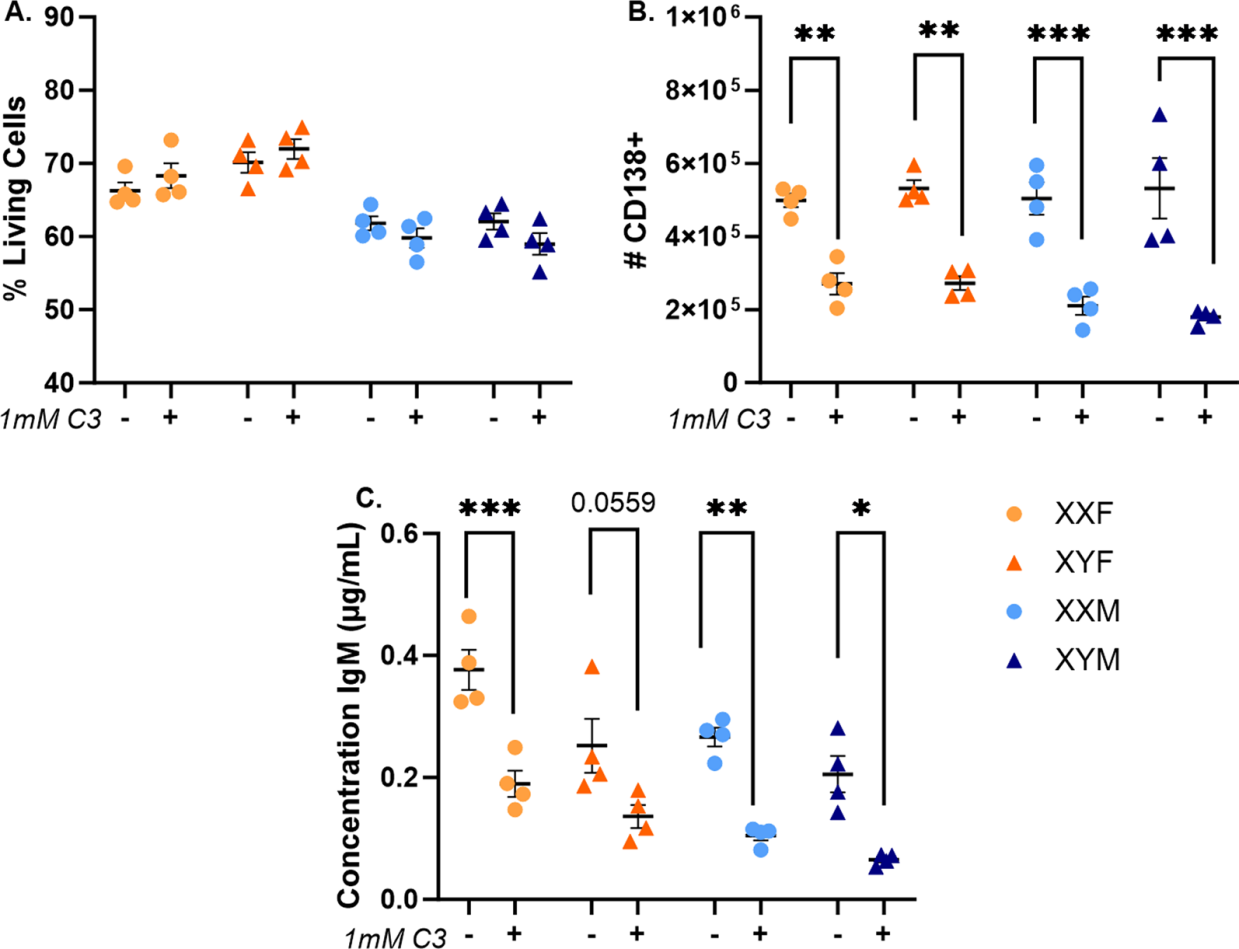
Propionate decreases plasma cell frequencies and IgM secretion similarly in all four genotypes ex vivo. Splenocytes isolated from naïve FCG mice were stimulated ex vivo with IL-4 (0.01 µg/mL) and LPS (5 µg/mL) in the presence or absence of 1 mM C3 (propionate). Flow cytometric analyses evaluated cell viability (**A**) and the number of CD138 + plasma cells (**B**) 4 days post-stimulation. Supernatants were collected and total IgM concentrations were assessed by ELISA (**C**). Data are represented as the mean ± SEM with each point representing one mouse. Statistics by three-way ANOVA with Tukey’s multiple comparisons test. ANOVA results provided in Additional file 9: Table S16. Comparisons in graphs are representative of multiple comparisons tests; *p < 0.05; **p < 0.01; ***p < 0.001; ****p < 0.0001. C3 = propionate. Additional concentrations of C3 are presented in Additional file 8: Fig. S8 with accompanying ANOVA table in Additional file 9: Table S17

Given the influence of gut-resident SCFA-producing bacteria on humoral immune responses in XX mice, we postulated that SCFAs could act directly on B cells to enhance their function in a sex-chromosome-dependent manner. To evaluate this possibility, splenocytes were isolated from FCG mice and stimulated ex vivo with LPS + IL-4 in the presence or absence of increasing, biologically relevant propionate (C3) concentrations (Additional file 8: Fig. S8). In addition to our previous research demonstrating that propanil impacted humoral immune responses in an XX-dependent manner, of the three most abundant SCFAs in the gut microbiome with known immunomodulatory function, propionate possesses moderate HDAC inhibition activity and presence in systemic circulation. Propionate exposure up to 1 mM did not alter cell viability following mitogenic stimulation. Interestingly, female cells demonstrated higher viability following ex vivo stimulation than male cells (*p* < 0.0001; Fig. 8A, Additional file 9: Table S16). Plasma cell differentiation was reduced in response to propionate treatment (*p* < 0.0001; Fig. 8B, Additional file 9: Table S16). Importantly, this reduction was similar in all genotypes and not affected by the sex chromosome complement (*p* = 0.5654), gonadal sex (*p* = 0.1256), or any two-way interactions (Additional file 9: Table S16). These impacts were dose-dependent and exerted effects similarly between the genotypes across multiple concentrations (Additional file 8: Fig. S8B, Additional file 9: Table S17). IgM production was similarly reduced in the presence of propionate (Fig. 8C, Additional file 8: Fig. S8C), for which all three variables (gonadal sex, chromosome complement, and treatment) were found to have significant effects (Additional file 9: Table S16). This is consistent with our previously presented observations that female cells produce more IgM than male cells and XX cells produce more IgM than XY cells ex vivo (Fig. 3, Additional file 9: Table S6). However, since the presence of propionate influenced XX and XY cells similarly ex vivo, we concluded that it is not influencing humoral responses in an XX-dependent manner in this context.

## Discussion

In the present study, humoral immune responses against HKSP immunization were found to be regulated not only by gonadal sex and their accompanying sex hormones, but also by the presence of an XX vs. XY sex chromosome complement (Fig. 1). Females produced significantly more HKSP-specific IgM-secreting cells than males, which was attributed to both gonadal sex, i.e., differential circulating sex hormones, and the sex chromosome complement (Additional file 9: Table S1). Differences in plasma cell generation, on the other hand, were only attributable to the sex chromosome complement and not gonadal sex (Additional file 9: Table S1), prompting us to further investigate mechanisms by which the sex chromosomes differentially contribute to immune activation.

A number of genetic factors could be contributing to the observed XX-dependent phenotypes. Among these are X-linked immune gene dosage effects, effects of genes encoded by the Y chromosome that are not expressed in XX mice [75], X gene parental imprinting, and the expression of X-linked microRNAs (miRNAs) [76, 77]. The current study focused on X-linked gene dosage effects. While the dosages of X-linked genes are typically balanced between males and females via the process of XCI [16], X-linked genes related to immunity have been demonstrated to more readily escape XCI in immune cells than other somatic cell types, and are uniquely regulated in B lymphocytes [17–21]. Furthermore, the biallelic expression of multiple X-linked, immune-related genes has recently been reported to promote lymphocyte activation [15, 16, 67, 68]. We therefore initially hypothesized that X-linked gene dosage effects may contribute to the sex chromosome-dependent phenotype we identified in response to HKSP immunization. RNA-Seq performed on splenocytes isolated from male and female FCG mice identified three X-linked genes, *Xist*, *Eif2s3x*, and *Kdm6a,* as being overexpressed in an XX vs. XY-dependent manner (Fig. 2A, Additional file 3: Fig. S3). The number of potential escape genes identified was lower than anticipated given the unique regulation of XCI in B cells but was consistent with previous reports suggesting that only 3% of X-linked genes escape inactivation in mouse embryonic fibroblasts. Higher percentages, upward of 15–20%, are believed to be biallelically expressed in human cells [18, 78]. While *Eif2s3x* is a commonly recognized escape gene, *Kdm6a* has only recently gained attention as an epigenetic modulator of sex-biased immune activation. Functioning as a histone H3 demethylase, *Kdm6a* has been linked to enhanced activation of female CD4 + T cell in EAE [68], as well as female microglial activation in ischemic stroke [67]. Although *Kdm6a* mutations have been associated with B cell cancers [79, 80], its impact on humoral responses to infection and vaccination are only beginning to be assessed. Conservation of *Kdm6a* overexpression in XX vs. XY immune cells across species (mouse and human) [81] and its epigenetic regulatory function made it an interesting target for additional studies attempting to delineate the mechanisms that contribute to more robust humoral responses in XX vs. XY mice.

Following RNA-Seq, subsequent experiments confirmed Kdm6a overexpression in XX vs. XY B cells at both the RNA and protein level (Fig. 2). Since overexpression does not necessarily equate to biallelic expression, RNA-FISH was utilized to examine whether *Kdm6a* is expressed from the inactive X chromosome. In these experiments, only 13% of the B cells isolated from HKSP immunized mice possessed detectable *Xist* clouds (Additional file 4: Fig. S4C), an indicator of X inactivation. Since *Xist* expression is low in naïve B cells and increases in response to both immunization in vivo or ex vivo stimulation (Additional file 4: Fig. S4E and [17, 71]), it was hypothesized that the B cells with detectable *Xist* clouds were reflective of activated B cell subsets (Additional file 4: Fig. S4). Of the B cells possessing *Xist* clouds, 78% demonstrated colocalization of *Kdm6a* and *Xist* signals suggesting that, similar to previous studies in mouse embryonic fibroblasts [81], *Kdm6a* can be expressed from the inactive X in a subset of B cells (Fig. 2H, Additional file 4: Fig. S4D). Interestingly, isolated plasma cells showed high frequencies of *Xist* clouds, but less *Xist/Kdm6a* colocalization (13%, Additional file 4: Fig. S4C-D), suggesting that the expression of *Kdm6a* from the inactive X chromosome may decrease after terminal differentiation into plasma cells. Due to the lack of *Xist* expression in naïve B cells, no conclusion could be drawn about whether *Kdm6a *is also biallelically expressed in this B cell subset in the current studies. Small nucleotide polymorphisms (SNPs) present in the maternal vs. paternal *Kdm6a* could be utilized to confirm *Kdm6a* biallelic expression in both naïve and activated B cell subsets. However, a different background strain would be needed, as C56BL6/J mice possessed no SNPs in their *Kdm6a* genes capable of distinguishing the maternal vs. paternal X chromosome.

*Kdm6a* overexpression has been demonstrated to promote T cell [19] and NK [82] function in a sex-dependent manner. In B cell cancers, it has been identified as a tumor suppressor [83] and, in recent studies, B cell activation, isotype switching, and plasma cell differentiation were restrained by KDM6a activity [84, 85], suggesting different functional roles in different lymphocyte populations. Adding to the existing B cell data, inhibition of KDM6a activity using GSK J4 increased plasma cell differentiation in all four genotypes (XXF, XYF, XXM, XYM) at high doses following ex vivo stimulation (Fig. 3, Additional file 5: Fig. S5). Furthermore, GSK J4-mediated induction of plasma cell differentiation was demonstrated to not be sex chromosome-dependent, as XX and XY cells exhibited similar sensitivities to GSK J4-mediated inhibition. It should be noted that the inhibitory function of GSK J4 is not limited to KDM6a alone, but also impacts all JMJD3 histone demethylases. However, other JMJD3 histone methylase family members, including *Kdm5c,* were expressed at equivalent levels in XX and XY FCG mouse B cells (data not shown). It is possible that KDM6a influences B cell activation and differentiation in an XX-dependent manner independent of its enzymatic activity. However, this was not assessed in the current study. Additionally, it should be noted that B cell activation in our GSK J4-mediated KDM6a inhibition studies was the result of mitogenic stimulation (Fig. 3) rather than crosslinking of an antigen-specific B cell receptor as in our in vivo studies (Fig. 1). While KDM6a may not impact mitogen-induced plasma cell differentiation and antibody secretion, perhaps it does impact antigen-dependent signaling pathways in an XX vs. XY-dependent manner. The fact that enhanced plasma cells frequencies in XX vs. XY mice were lost in ex vivo studies could be suggestive that this is the case. However, an alternate hypothesis is that additional extrinsic factors in the in vivo environment can differentially regulate B cell activation in an XX-dependent manner. Given that sex hormones did not impact enhanced CD138 + plasma cell frequencies in XX vs. XY mice, and our previous work with propanil, we hypothesized that the SCFA-producing bacteria in the gut may impact immune activation in a sex chromosome-dependent manner.

The gut microbiome has more recently been established as an important regulator of immunity and has been demonstrated to differentially modulate immune activation in males and females [30–33]. To our knowledge, the collaborative role of the gut microbiome and XX sex chromosome complement has not previously been evaluated. Antibiotic depletion of the gut microbiome reduced humoral responses in XX mice to levels similar to that of XY, while having no impact on the magnitude of XY responses (Fig. 4, Additional file 9: Tables S8 and S9), suggesting the collaborative interaction of the gut microbiome and sex chromosome complement in regulating immune activation. Consistent with previous reports [86–88], sex-specific differences were noted in the composition of male vs. female gut microbiota (Fig. 5). However, minimal differences were identified in the microbiota abundancies of XXF vs. XYF females or XXM vs. XYM males (Fig. 5E), or in the levels of SCFAs between XX vs. XY mice of the same gonadal sex (Fig. 6). This led us to consider other mechanisms by which the gut microbiome could modulate immune responses in an XX-dependent manner. We previously demonstrated XX-specific immune enhancement by the immunomodulatory compound propanil [34], which is metabolized into the SCFA propionate. Building upon these previous findings, we hypothesized that gut microbiota-generated propionate may function in a similar manner to enhance B cell function in an XX-dependent manner.

To investigate this possibility, we depleted the endogenous gut bacteria of FCG mice with antibiotics and then reconstituted it with known SCFA-producing species prior to HKSP immunization. A significant increase in the number of ASC cells was observed in XX mice possessing microbiomes reconstituted with SCFA-producing bacteria compared to controls, while similar reconstitution had no impact on XY responses (Fig. 7D, E). This phenotype held true for both females and males, and although females saw better colonization of SCFA-producing species than males (Fig. 7B, C), similar to data generated using microbiome-intact animals (Fig. 6), males had increased levels of fecal SCFAs (Fig. 7F–H, Additional file 7: Fig. S7, and Additional file 9: Table S15). Interestingly, the main effect of gonadal sex, in which females responded more robustly to HKSP immunization than males (Fig. 1), was lost in mice whose microbiota were depleted and reconstituted with SCFA-producing species (Additional file 9: Table S14). Taken together, these data suggest that sex-specific gut microbiome compositions may influence the robustness of an immune response. However, those compositional differences alone are not mediating the XX sex chromosome-dependent effects noted following HKSP immunization, and thus requires additional study.

One manner in which the SCFA-producing bacteria influence immunity is by acting as histone deacetylase (Class I/II) inhibitors [40, 42, 89–92]. In B cells, SCFAs have been demonstrated to inhibit HDAC activity, resulting in histone H3 hyper-acetylation, plasma cell differentiation, and class-switching [40]. Subsequent opposing studies have alternatively suggested that SCFAs both promote and suppress B cell responses, depending upon the exposure dose [40, 93]. In order to confirm the results of our in vivo studies demonstrating that gut-resident SCFA-producing bacteria modulate immune activation in a sex chromosome-dependent manner, and to establish a model capable of evaluating the influence of HDAC inhibition on the identified XX-dependent phenotype, we evaluated the influence of propionate on XX vs. XY B cell activation in response to ex vivo mitogenic stimulation. Concentrations utilized were similar to those previously reported by Kim et al*.* [40] and Sanchez et al*.* [93]. In contrast to our in vivo studies, which demonstrated elevated numbers of antigen-specific IgM-secreting cells in XX animals when SCFA-producing bacteria were present (Fig. 7), but in concurrence with Sanchez et al*.,* propionate decreased mitogen-induced plasma cell differentiation in a dose-dependent manner (Fig. 8, Additional file 8: Fig S8). Importantly, it did so similarly in all four genotypes, suggesting that propionate may not be directly contributing to the enhanced XX immune responses observed in vivo. However, it is important to note that additional sex-specific collaborations occur in our in vivo studies that may not be present in our ex vivo studies, which could implicate the organizational or activational influences of sex hormones on the gut microbiome-mediated effects. For example, while sex hormones are able to impact microbiome compositions (Fig. 5 and [86–88, 94]), gut bacteria have also been demonstrated to influence the sex hormones concentrations or bioavailability [30, 31]. Such effects would be absent in our ex vivo studies. In addition, as mentioned above in our GSK J4 ex vivo studies, B cell activation in our in vivo vs. ex vivo studies utilized distinct signal transduction pathways to induce B cell activation, which could complicate the interpretation of these studies. Taken together, these data suggest that SCFA-producing bacteria in the gut microbiome influence immune responses in an XX-specific manner, but the underlying mechanisms by which they do so need further investigation.

While our study provides valuable insights into the complex interactions between the sex chromosome complement and gut microbiome in shaping immune responses, it is essential to acknowledge the limitations of the FCG mouse model. Others have demonstrated similar levels of circulating sex hormones in XX vs. XY mice of the same gonadal sex [28, 95–97], but variations in gonadal morphology and function between XX and XY mice of the same gonadal sex, as well as potential differences in the phenotypic responses to cyclic ovarian hormones [98, 99], should be considered when interpreting our findings. We attempted to control for these variables by gonadectomy when possible, but the potential for hormone organizational effects prior to gonadectomy cannot be overlooked. Additionally, the variability in gut microbiome composition observed between animal facilities is a challenge whenever studying its role in biological processes. We attempted to mitigate this challenge by depleting the endogenous microbiome and reconstituting with select species, which may be a valuable strategy for obtaining reproducible results in multiple settings.

### Perspectives and significance

While the individual impacts of sex hormones, sex chromosome complement, and the gut microbiome on immunity have been well characterized [1, 37, 100–102], the present study underscores the essential consideration that these three biological systems are intrinsically interconnected. The collaboration between sex hormones and sex chromosomes has previously been evaluated, but less is known about the interplay between these sex-specific factors and the gut microbiome. Sex hormones are known to be crucial regulators of microbiome colonization [30, 31, 33], and conversely, the gut microbiota can influence hormone production and bioactivity [32]. Most previous studies investigating microbiome-dependent influences have focused on identifying sex-specific microbiome populations to explain dimorphic responses. However, in the current studies, while attempting to delineate the underlying mechanisms contributing to sex biases in immune responses to HKSP immunization, we demonstrated that similar gut microbiomes can influence immune sex biases in an XX sex chromosome-dependent manner.

### Supplementary Information

**Additional file 1: Figure S1. **Four Core Genotype breeding strategy and offspring genotypes.

**Additional file 2: Figure S2. **Experimental design of experiments evaluating whether the gut microbiota contributes to XX-dependent immune enhancement following HKSP immunization.

**Additional file 3: Figure S3. **RNA-Sequencing to identify X-linked genes overexpressed in XX vs. XY splenocytes. Volcano plots depicting genes identified as under-expressed (blue), expressed similarly (black), or overexpressed in XX vs. XY females (A) and males (B) using the threshold of log2FC > 0.585 and FDR < 0.1. Three X-linked genes were identified as overexpressed in female and male XX vs. XY cells (*Xist,* *Eif2s3x,* and *Kdm6a*) and, along with the Y-linked gene *Uty*, are labeled in the volcano plots. Genes expected to be identified as differentially expressed in females vs. males and in XX vs. XY FCG mice by RNA sequencing were graphed as EdgeR expression values to validate sequencing data. *Xist* is a long, non-coding RNA expressed from the inactive X chromosome and therefore only in cells possessing an XX sex chromosome complement (A). *Uty* is a Y-linked homolog of *Kdm6a* and should only be expressed in cells possessing a Y chromosome (B). *Sry* is the testes-determining gene and should only be expressed in gonadal males (XXM and XYM), and not in gonadal XXF or XYF females (E). Data are represented as the mean ± SEM with each data point representing one mouse. Statistics by two-way ANOVA with Tukey’s multiple comparisons test. Comparisons in graphs are representative of multiple comparisons tests; ***p < 0.001; ****p < 0.0001. ANOVA main and interactive effects are provided in Additional file 9: Table S5.

**Additional file 4: Figure S4. ***Xist* expression and colocalization with *Kdm6a* in total B cells and plasma cells. RNA-FISH was performed in total B cells (A) and isolated plasma cells (B) from the spleens of HKSP-immunized XXF mice. Arrow in A indicates colocalization of *Xist* and *Kdm6a*, while arrows in B indicate *Xist* expression without *Kdm6a* colocalization. The number of cells exhibiting *Xist* points were quantified in each (C). Of cells exhibiting *Xist* points, the cells demonstrating colocalization of *Kdm6a* with *Xist* were quantified (D). Splenocytes isolated from naïve XXF mice were stimulated ex vivo with IL-4 (0.01 µg/mL) and LPS (5 µg/mL) and cells collected at indicated time points for RNA isolation to evaluate *Xist* expression relative to *Gapdh* by qRT-PCR (E). Data in C and D are represented as the mean ± SEM. Statistics by unpaired t-tests; **p < 0.01; ****p < 0.0001

**Additional file 5: Figure S5. **Impact of GSK J4 and inactive isomer GSK J5 on ex vivo plasma cell frequencies and IgM production. Splenocytes isolated from naïve FCG mice were stimulated ex vivo with IL-4 (0.01 µg/mL) and LPS (5 µg/mL) in the presence or absence of GSK J4 (A-B) or GSK J5 (C-D) at additional concentrations supplemental to Fig. 3. Percentages of CD138 + plasma cells in FCG mice were quantified by flow cytometry (A, C). Supernatants were collected and total IgM concentrations were assessed by ELISA for each stimulation condition (B, D). Data are represented as the mean ± SEM with each data point representing one mouse. Statistics by three-way ANOVA are provided in Additional file 9: Table S7.

**Additional file 6: Figure S6. **Confirmation of microbiome depletion and reconstitution. Representative images of flow plots showing dead and viable bacteria in fecal pellets collected during the experiment depicted in Fig. 7. Fecal pellets were collected from one mouse per genotype in each experimental treatment group pre-antibiotics (A, Intact Microbiome, Day 0), post-antibiotics/pre-treatment with SCFA-producers ± inulin (B, Depleted Microbiome, Day 4), and at experimental end point (C-E, Day 13).

**Additional file 7: Figure S7.** Concentrations of additional SCFA measured in the fecal pellets of FCG mice following depletion and reconstitution of SCFA-producing bacteria. Following antibiotic depletion (Days 1–3) and SCFA-producing bacteria reconstitution (Days 4–5) in Fig. 7, fecal pellets were collected from the inulin alone mice (-) and the inulin + SCFA-producers mice ( +) on Day 13, the final day of the experiment. Concentrations of the following additional SCFAs were assessed by LC–MS/MS (Metabolon): 2-methylbutyric acid (A), hexanoic acid (caproic acid, B), isobutyric acid (C), and isovaleric acid (D). Data are represented as the mean ± SEM with each point representing one mouse. ANOVA main and interactive effects are provided in Additional file 9: Table S15.

**Additional file 8: Figure S8. **Impact of additional propionate (C3) concentrations on responses to mitogenic stimulation ex vivo. Splenocytes isolated from naïve FCG mice were stimulated ex vivo with IL-4 (0.01 µg/mL) and LPS (5 µg/mL) in the presence or absence of C3 (propionate) at additional concentrations supplemental to Fig. 8. Flow cytometric analyses evaluated cell viability (A) and the number of CD138 + plasma cells (B) 4 days post-stimulation. Supernatants were collected and total IgM concentrations were assessed by ELISA (C). Data are represented as the mean ± SEM. Statistics by three-way ANOVA with Tukey’s multiple comparisons test. Comparisons in graphs are representative of multiple comparisons tests; *p < 0.05; **p < 0.01; ***p < 0.001; ****p < 0.0001. C3 = propionate. Statistics by three-way ANOVA are provided in Additional file 9: Table S17.

**Additional file 9.** Supplementary Tables.

## Data Availability

RNA sequencing has been deposited to GEO (accession number GSE244866) with the following secure token generated for reviewers’ purposes: https://www.ncbi.nlm.nih.gov/geo/query/acc.cgi?acc=GSE244866. Raw sequencing data for 16 s rRNA sequencing are available at PRJNA1023556 in the NCBI SRA database. Other data generated and/or analyzed during the current study are available from the corresponding author on reasonable request.

## Abbreviations

FCG: Four Core Genotype
HKSP: Heat-killed *Streptococcus pneumoniae*
XCI: X chromosome inactivation
SCFA: Short-chain fatty acids
EAE: Experimental autoimmune encephalomyelitis
SLE: Systemic lupus erythematosus
RNA-FISH: RNA fluorescent in situ hybridization
Gdx: Gonadectomized
ASC: Antibody secreting cell
OTUs: Observed taxonomic units
