# Supplementary figures and images for: XX sex chromosome complement modulates immune responses to heat-killed Streptococcus pneumoniae immunization in a microbiome-dependent manner
Source: Biol Sex Differ. 2024 Mar 14;15:21. doi: 10.1186/s13293-024-00597-0 (PMC10938708; doi:10.1186/s13293-024-00597-0)

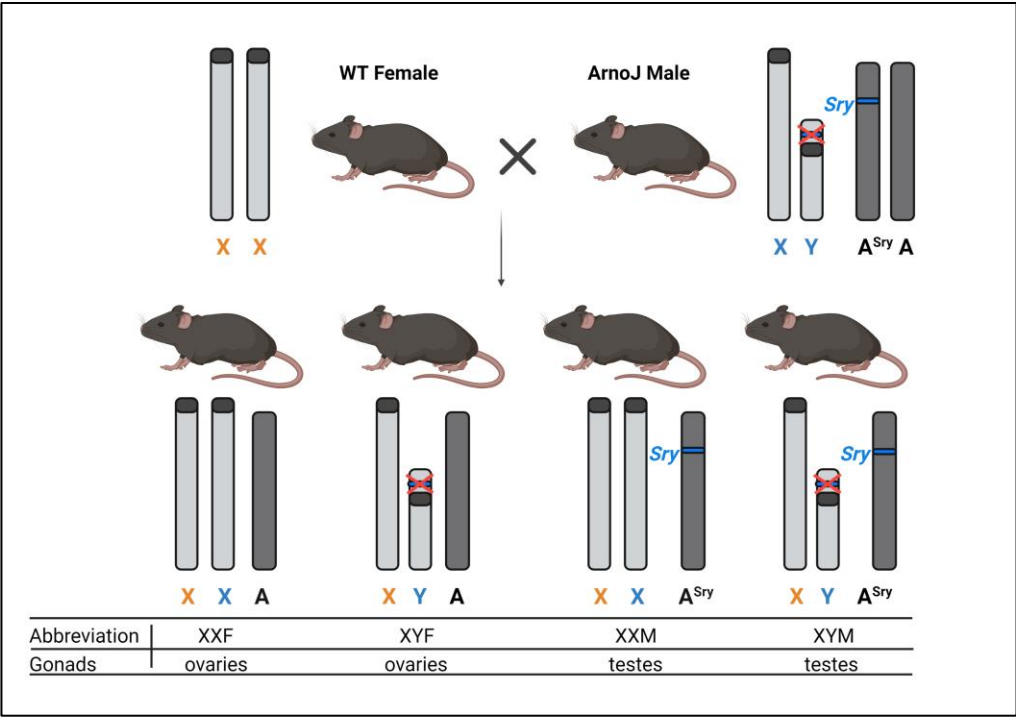

Supplement: Supplementary file 1 — Additional file 1: Figure S1. Four Core Genotype breeding strategy and offspring genotypes. [file 13293_2024_597_MOESM1_ESM.pdf]

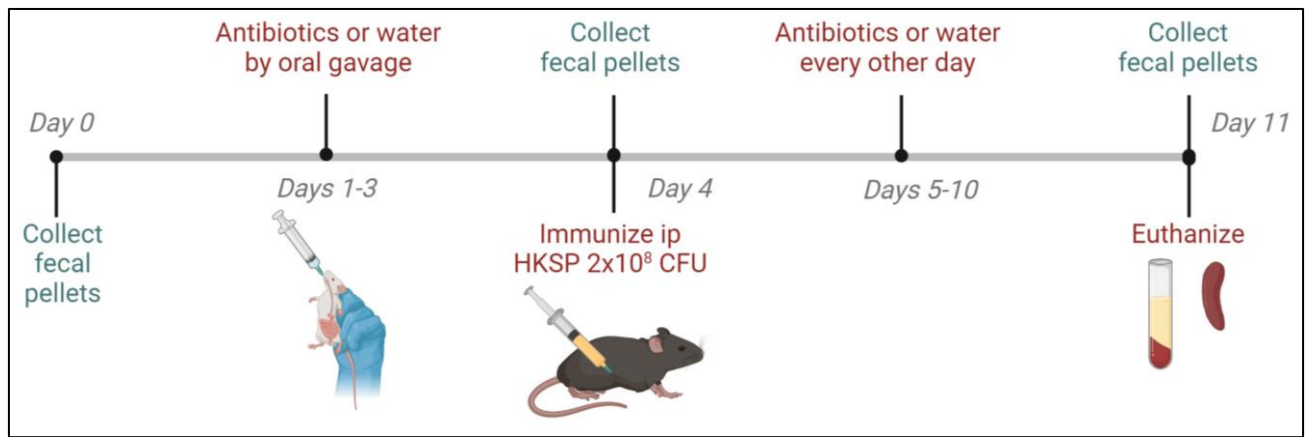

Supplement: Supplementary file 2 — Additional file 2: Figure S2. Experimental design of experiments evaluating whether the gut microbiota contributes to XX-dependent immune enhancement following HKSP immunization. [file 13293_2024_597_MOESM2_ESM.pdf]

## Slide 1
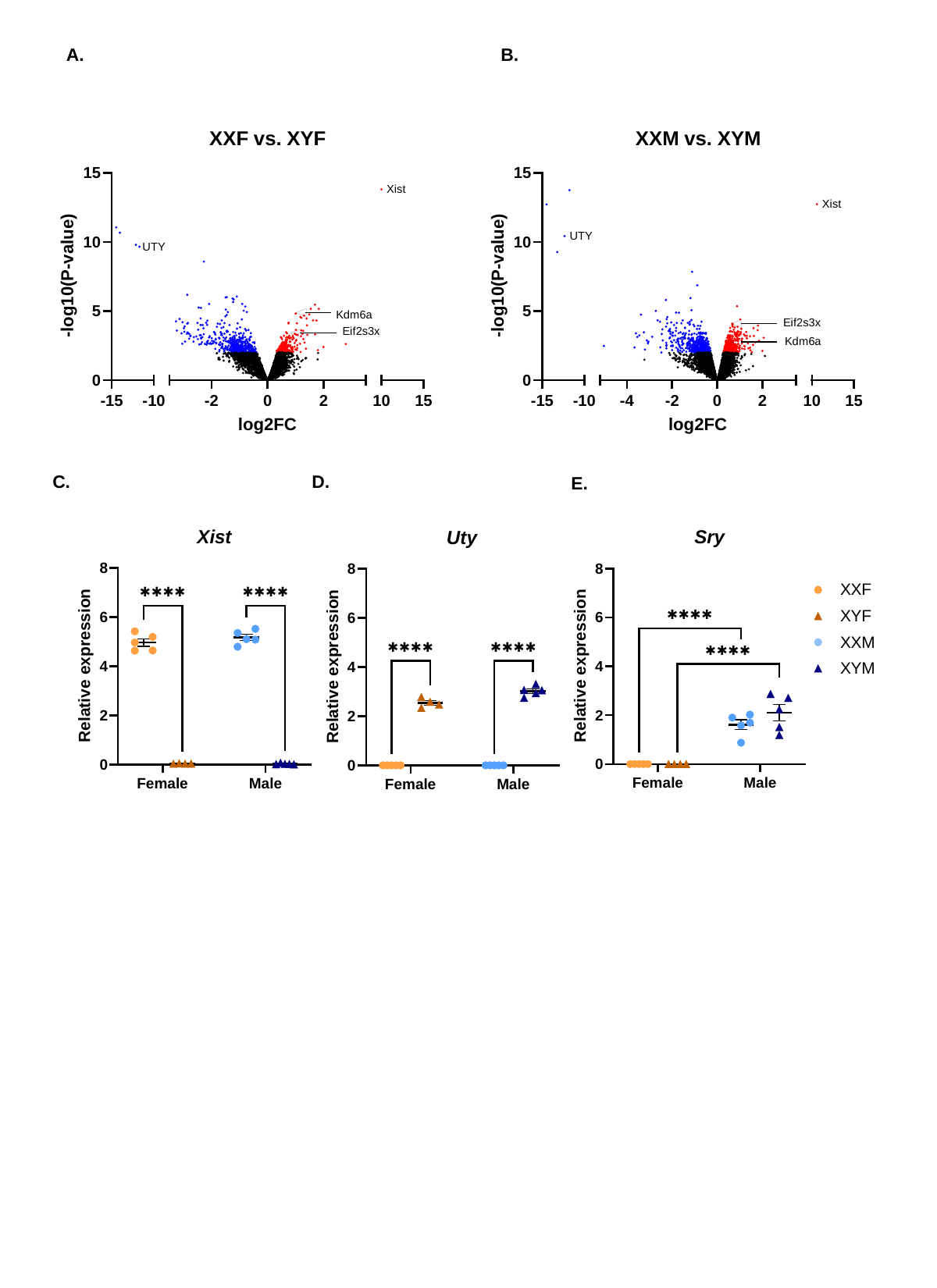

A.
B.
C.
D.
E.

Supplement: Supplementary file 3 — Additional file 3: Figure S3. RNA-Sequencing to identify X-linked genes overexpressed in XX vs. XY splenocytes. Volcano plots depicting genes identified as under-expressed (blue), expressed similarly (black), or overexpressed in XX vs. XY females (A) and males (B) using the threshold of log2FC > 0.585 and FDR < 0.1. Three X-linked genes were identified as overexpressed in female and male XX vs. XY cells (Xist, Eif2s3x, and Kdm6a) and, along with the Y-linked gene Uty, are labeled in the volcano plots. Genes expected to be identified as differentially expressed in females vs. males and in XX vs. XY FCG mice by RNA sequencing were graphed as EdgeR expression values to validate sequencing data. Xist is a long, non-coding RNA expressed from the inactive X chromosome and therefore only in cells possessing an XX sex chromosome complement (A). Uty is a Y-linked homolog of Kdm6a and should only be expressed in cells possessing a Y chromosome (B). Sry is the testes-determining gene and should only be expressed in gonadal males (XXM and XYM), and not in gonadal XXF or XYF females (E). Data are represented as the mean ± SEM with each data point representing one mouse. Statistics by two-way ANOVA with Tukey’s multiple comparisons test. Comparisons in graphs are representative of multiple comparisons tests; ***p < 0.001; ****p < 0.0001. ANOVA main and interactive effects are provided in Additional file 9: Table S5. [file 13293_2024_597_MOESM3_ESM.pptx]

## Slide 1
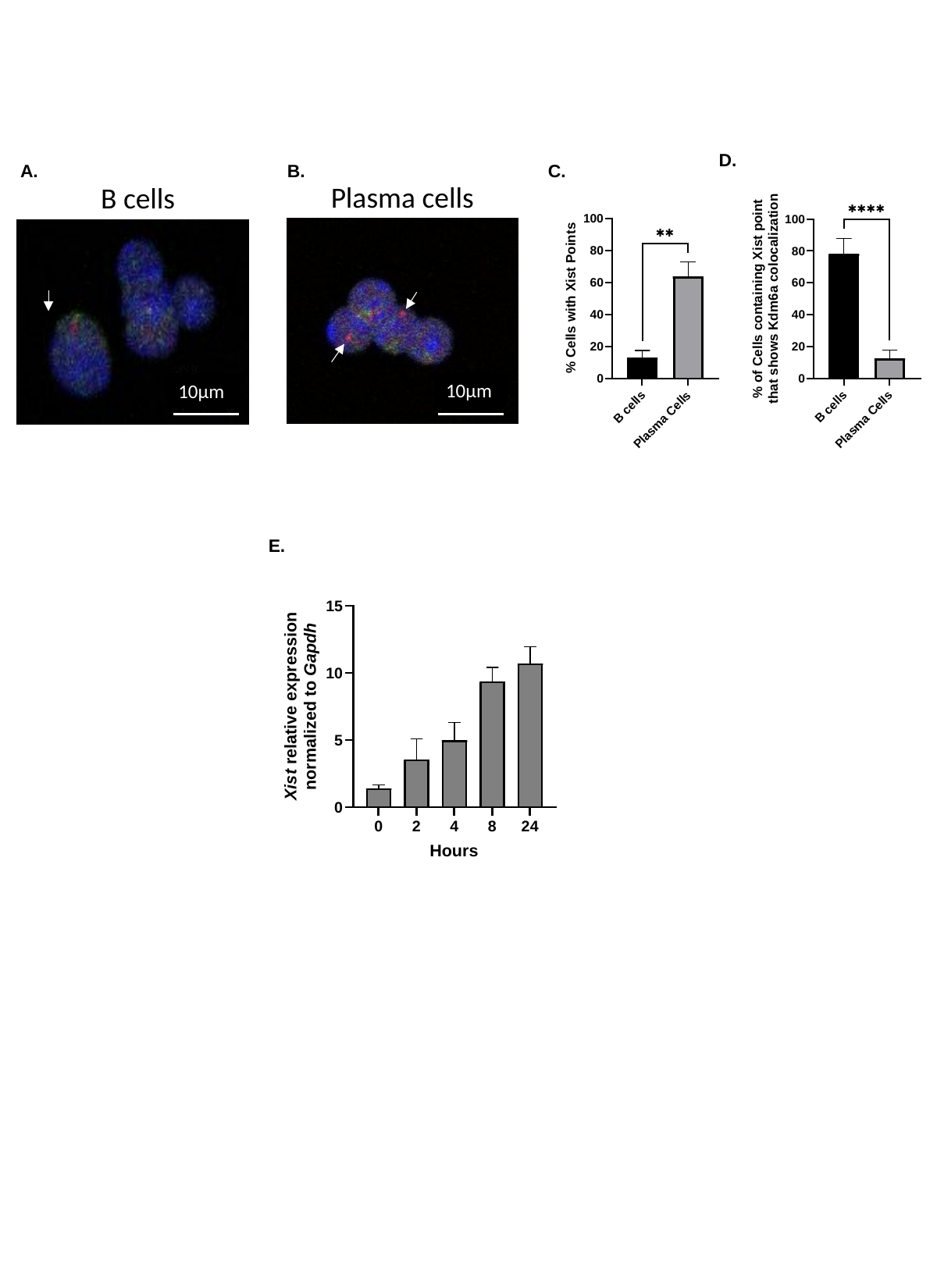

B.
C.
D.
A.
Plasma cells
10µm
B cells
10µm
E.

Supplement: Supplementary file 4 — Additional file 4: Figure S4. Xist expression and colocalization with Kdm6a in total B cells and plasma cells. RNA-FISH was performed in total B cells (A) and isolated plasma cells (B) from the spleens of HKSP-immunized XXF mice. Arrow in A indicates colocalization of Xist and Kdm6a, while arrows in B indicate Xist expression without Kdm6a colocalization. The number of cells exhibiting Xist points were quantified in each (C). Of cells exhibiting Xist points, the cells demonstrating colocalization of Kdm6a with Xist were quantified (D). Splenocytes isolated from naïve XXF mice were stimulated ex vivo with IL-4 (0.01 µg/mL) and LPS (5 µg/mL) and cells collected at indicated time points for RNA isolation to evaluate Xist expression relative to Gapdh by qRT-PCR (E). Data in C and D are represented as the mean ± SEM. Statistics by unpaired t-tests; **p < 0.01; ****p < 0.0001 [file 13293_2024_597_MOESM4_ESM.pptx]

## Slide 1
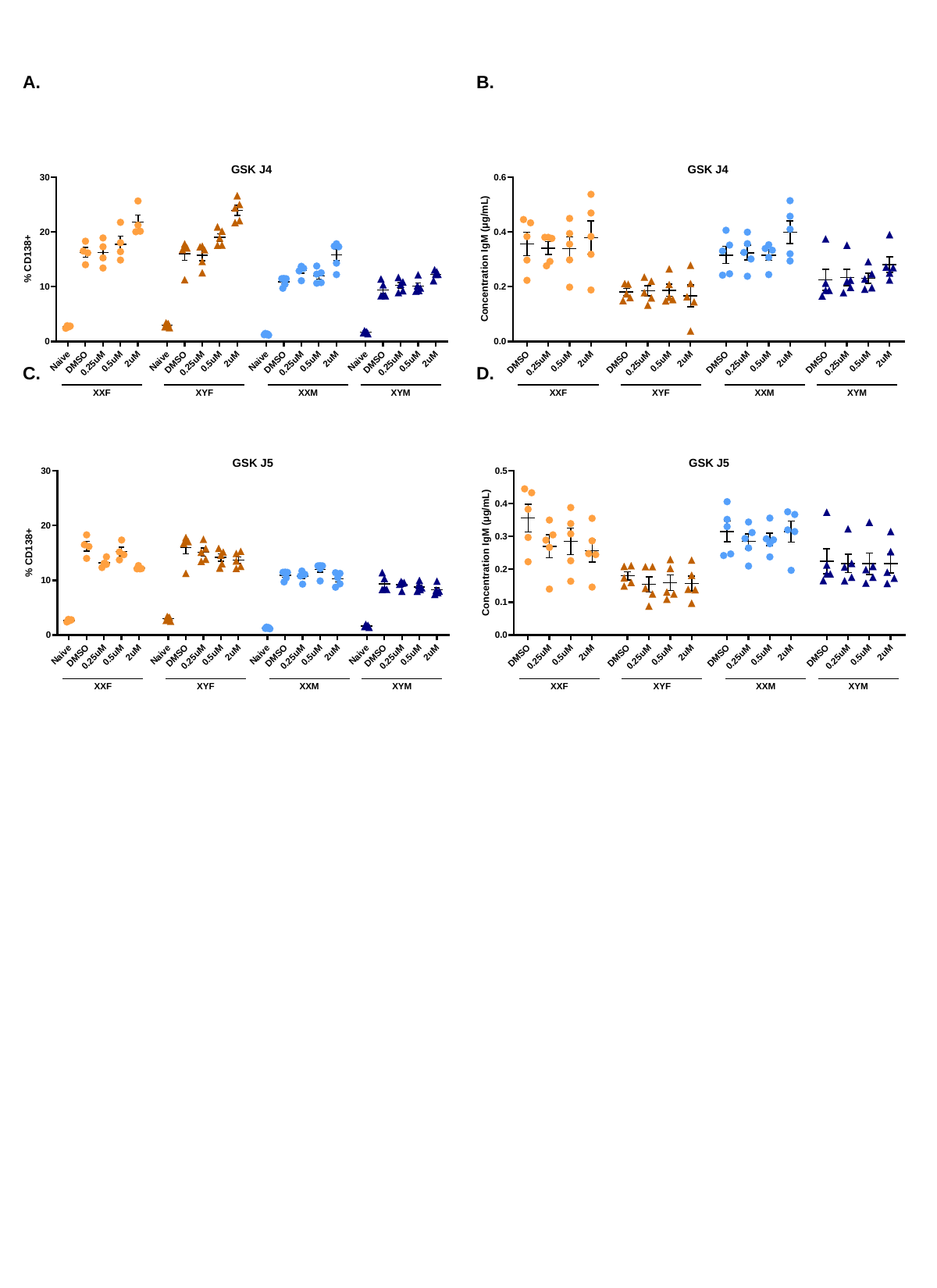

B.
A.
D.
C.

Supplement: Supplementary file 5 — Additional file 5: Figure S5. Impact of GSK J4 and inactive isomer GSK J5 on ex vivo plasma cell frequencies and IgM production. Splenocytes isolated from naïve FCG mice were stimulated ex vivo with IL-4 (0.01 µg/mL) and LPS (5 µg/mL) in the presence or absence of GSK J4 (A-B) or GSK J5 (C-D) at additional concentrations supplemental to Fig. 3. Percentages of CD138 + plasma cells in FCG mice were quantified by flow cytometry (A, C). Supernatants were collected and total IgM concentrations were assessed by ELISA for each stimulation condition (B, D). Data are represented as the mean ± SEM with each data point representing one mouse. Statistics by three-way ANOVA are provided in Additional file 9: Table S7. [file 13293_2024_597_MOESM5_ESM.pptx]

## Slide 1
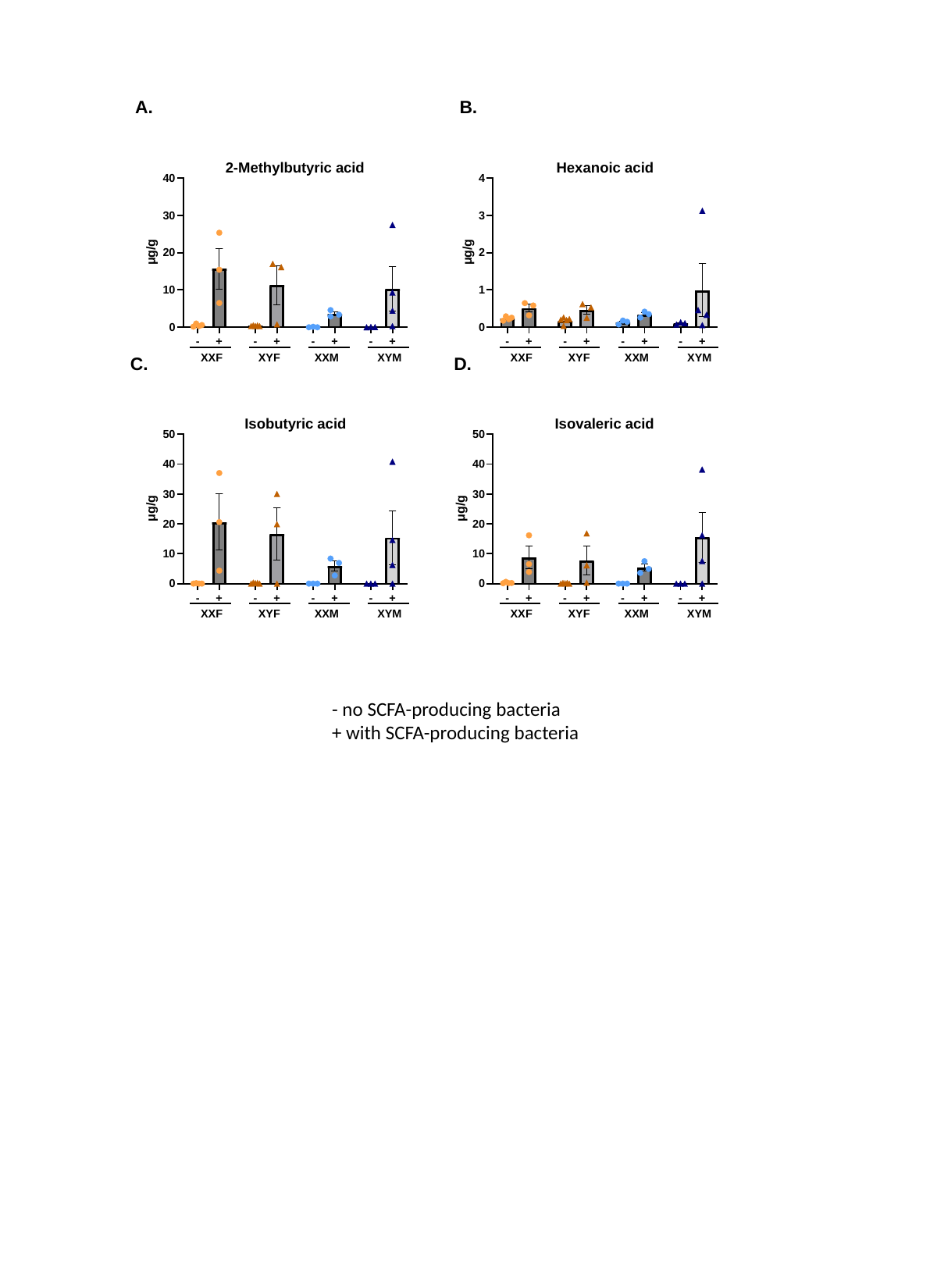

B.
A.
C.
D.
- no SCFA-producing bacteria
+ with SCFA-producing bacteria

Supplement: Supplementary file 7 — Additional file 7: Figure S7. Concentrations of additional SCFA measured in the fecal pellets of FCG mice following depletion and reconstitution of SCFA-producing bacteria. Following antibiotic depletion (Days 1–3) and SCFA-producing bacteria reconstitution (Days 4–5) in Fig. 7, fecal pellets were collected from the inulin alone mice (-) and the inulin + SCFA-producers mice ( +) on Day 13, the final day of the experiment. Concentrations of the following additional SCFAs were assessed by LC–MS/MS (Metabolon): 2-methylbutyric acid (A), hexanoic acid (caproic acid, B), isobutyric acid (C), and isovaleric acid (D). Data are represented as the mean ± SEM with each point representing one mouse. ANOVA main and interactive effects are provided in Additional file 9: Table S15. [file 13293_2024_597_MOESM7_ESM.pptx]

## Slide 1
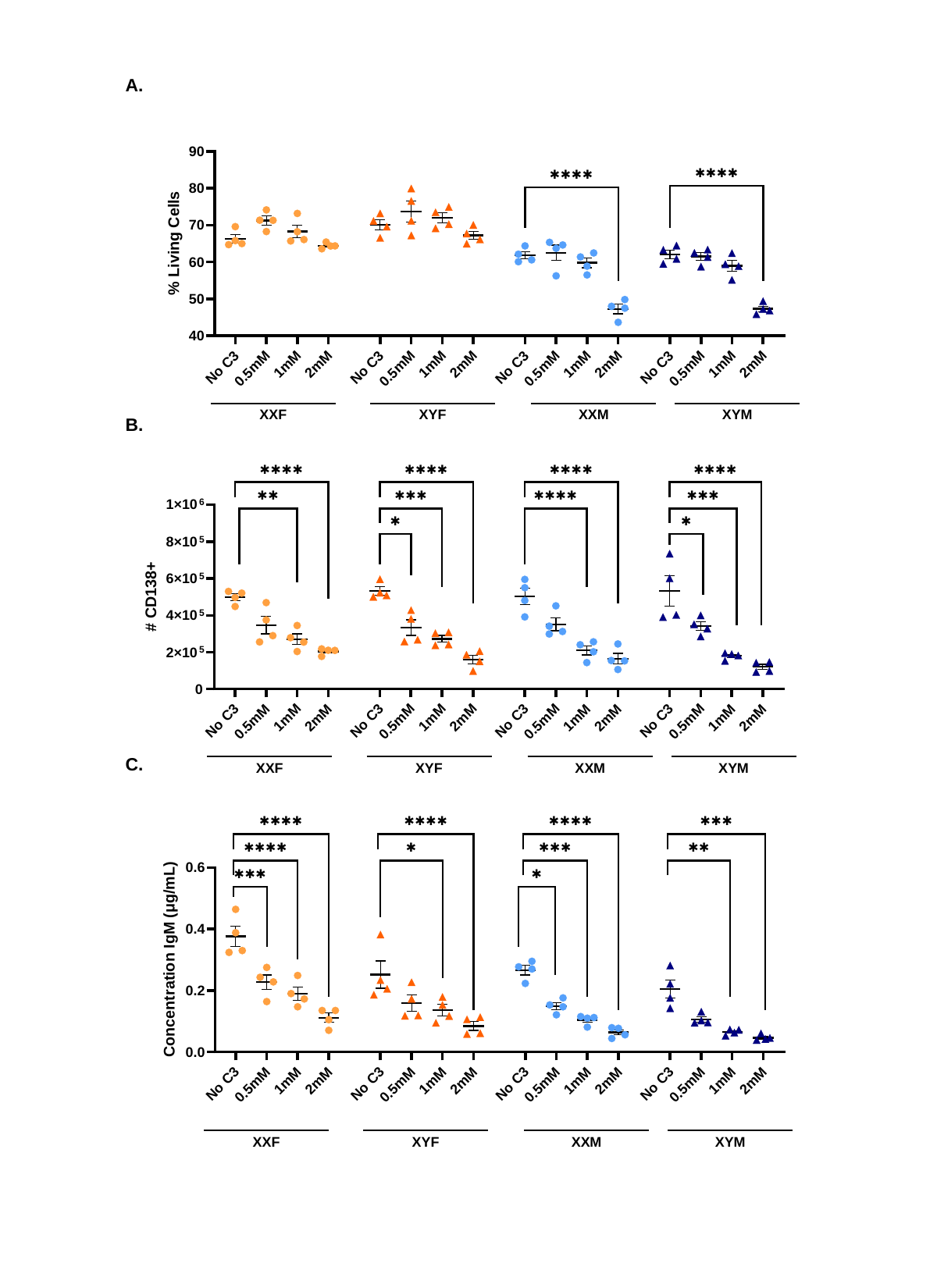

A.
B.
C.

Supplement: Supplementary file 8 — Additional file 8: Figure S8. Impact of additional propionate (C3) concentrations on responses to mitogenic stimulation ex vivo. Splenocytes isolated from naïve FCG mice were stimulated ex vivo with IL-4 (0.01 µg/mL) and LPS (5 µg/mL) in the presence or absence of C3 (propionate) at additional concentrations supplemental to Fig. 8. Flow cytometric analyses evaluated cell viability (A) and the number of CD138 + plasma cells (B) 4 days post-stimulation. Supernatants were collected and total IgM concentrations were assessed by ELISA (C). Data are represented as the mean ± SEM. Statistics by three-way ANOVA with Tukey’s multiple comparisons test. Comparisons in graphs are representative of multiple comparisons tests; *p < 0.05; **p < 0.01; ***p < 0.001; ****p < 0.0001. C3 = propionate. Statistics by three-way ANOVA are provided in Additional file 9: Table S17. [file 13293_2024_597_MOESM8_ESM.pptx]
