# Supplementary material for: XX sex chromosome complement modulates immune responses to heat-killed Streptococcus pneumoniae immunization in a microbiome-dependent manner
Source: Biol Sex Differ. 2024 Mar 14;15:21. doi: 10.1186/s13293-024-00597-0 (PMC10938708; doi:10.1186/s13293-024-00597-0)
Supplement: Supplementary file 6 — Additional file 6: Figure S6. Confirmation of microbiome depletion and reconstitution. Representative images of flow plots showing dead and viable bacteria in fecal pellets collected during the experiment depicted in Fig. 7. Fecal pellets were collected from one mouse per genotype in each experimental treatment group pre-antibiotics (A, Intact Microbiome, Day 0), post-antibiotics/pre-treatment with SCFA-producers ± inulin (B, Depleted Microbiome, Day 4), and at experimental end point (C-E, Day 13). [file 13293_2024_597_MOESM6_ESM.pptx]

## Slide 1
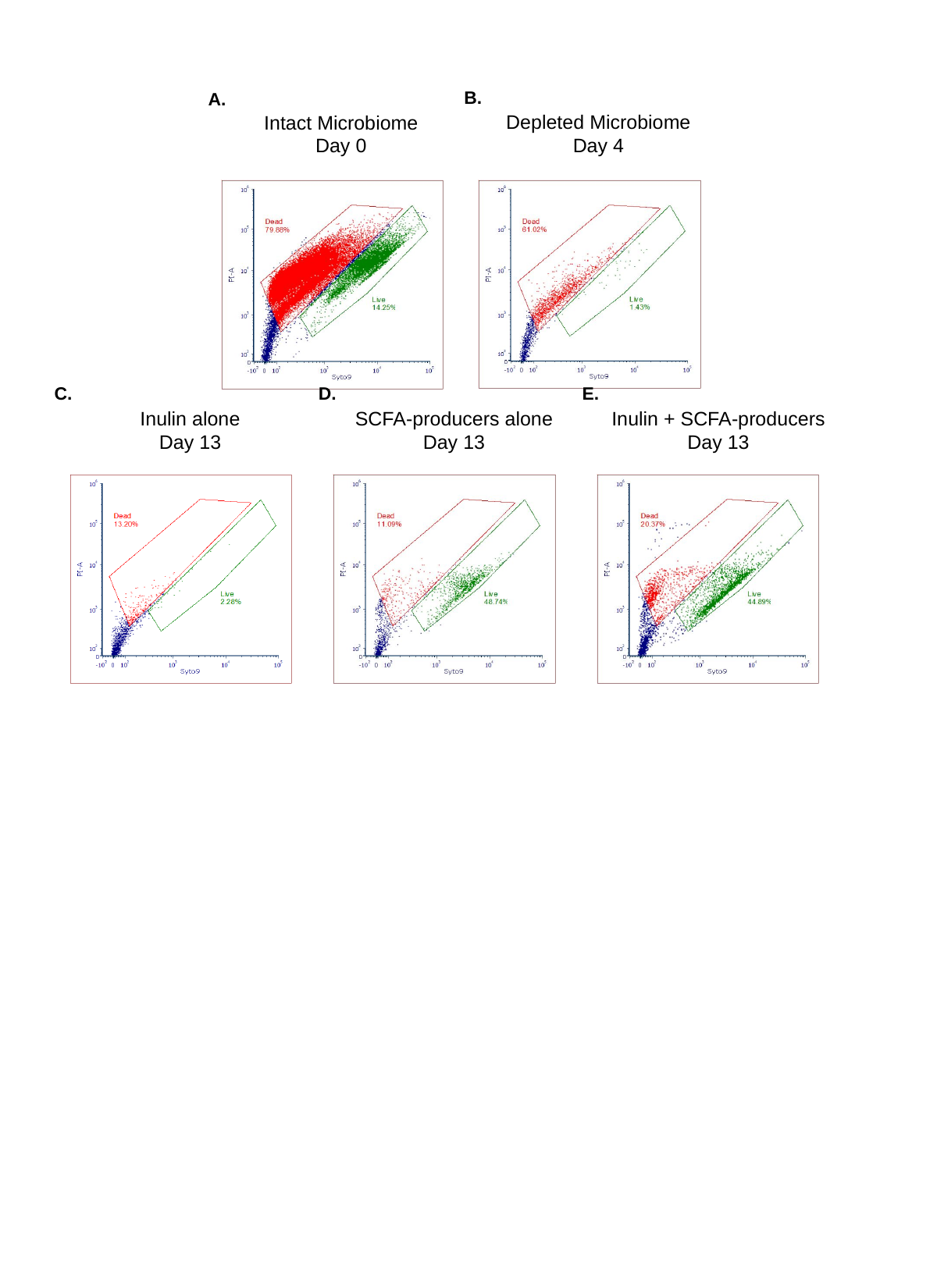

Depleted Microbiome
Day 4
Intact Microbiome
Day 0
Inulin alone
Day 13
SCFA-producers alone
Day 13
Inulin + SCFA-producers
Day 13
B.
A.
C.
D.
E.
