# Supplementary material for: XX sex chromosome complement modulates immune responses to heat-killed Streptococcus pneumoniae immunization in a microbiome-dependent manner
Source: Biol Sex Differ. 2024 Mar 14;15:21. doi: 10.1186/s13293-024-00597-0 (PMC10938708; doi:10.1186/s13293-024-00597-0)
Supplement: Supplementary file 9 — Additional file 9. Supplementary Tables. [file 13293_2024_597_MOESM9_ESM.docx]

**Table S1: Two-way ANOVA Fig. 1A-B**

|  | ***Fig. 1A***  ***IgM-secreting B cells*** | | ***Fig. 1B***  ***CD138+ plasma cells*** | |
| --- | --- | --- | --- | --- |
| ***Effect*** | ***F*** | ***p value*** | ***F*** | ***p value*** |
| *Main Effects* |  |  |  |  |
| Gonadal Sex (female, male) | **39.98** | **<0.0001** | 2.565 | 0.1258 |
| Chromosomes (XX, XY) | **13.78** | **0.0006** | **12.84** | **0.0020** |
| *Two-way Interaction* |  |  |  |  |
| Gonadal Sex*Chromosomes | **9.789** | **0.0032** | 0.3851 | 0.5423 |

*Summary of the two-way ANOVA results of main effects of gonadal sex and sex chromosomes and their interactions on the number of IgM-secreting B cells and CD138+ plasma cells one-week post-HKSP immunization shown in Fig. 1A-B. Bold values indicate significance (p*<0.05).

**Table S2: Three-way ANOVA Fig. 1C**

| ***Effect*** | ***F*** | ***p value*** |
| --- | --- | --- |
| *Main Effects* |  |  |
| Gonadal Sex (female, male) | 2.160 | 0.1508 |
| Chromosomes (XX, XY) | **31.04** | **<0.0001** |
| Gonads (Sham, Gdx) | **15.20** | **0.0004** |
| *Two-way Interactions* |  |  |
| Gonadal Sex*Chromosomes | **4.597** | **0.0393** |
| Gonadal Sex*Gonads | 0.6648 | 0.4205 |
| Chromosomes*Gonads | 1.447 | 0.2373 |
| *Three-way Interactions* |  |  |
| Gonadal Sex*Chromosomes*Gonads | 0.01854 | 0.8925 |

*Summary of the three-way ANOVA results of main effects of gonadal sex, sex chromosomes, and gonadal status (Gonads), and their interactions on the number of CD138+ plasma cells one-week post-HKSP immunization shown in Fig. 1C. Bold values indicate significance (p*<0.05).

**Table S3: Two-way ANOVA Fig. 2A&E**

|  | ***Fig. 2A.***  ***RNA-Seq*** | | ***Fig. 2E***  ***Protein*** | |
| --- | --- | --- | --- | --- |
| ***Effect*** | ***F*** | ***p value*** | ***F*** | ***p value*** |
| *Main Effects* |  |  |  |  |
| Gonadal Sex (female, male) | **5.589** | **0.0320** | 0.7316 | 0.4172 |
| Chromosomes (XX, XY) | **54.67** | **<0.0001** | **12.64** | **0.0075** |
| *Two-way Interactions* |  |  |  |  |
| Gonadal Sex*Chromosomes | **8.025** | **0.0126** | 0.1988 | 0.6675 |

*Summary of the two-way ANOVA results of main effects of gonadal sex and sex chromosomes and their interactions on Kdm6a expression and protein levels one-week post-HKSP immunization shown in Fig. 2A&E. Bold values indicate significance (p<0.05).*

**Table S4: Two-way ANOVA Fig. 2F-G**

|  | ***Fig. 2F***  ***Females*** | | ***Fig. 2G***  ***Males*** | |
| --- | --- | --- | --- | --- |
| ***Effect*** | ***F*** | ***p value*** | ***F*** | ***p value*** |
| *Main Effects* |  |  |  |  |
| Gonadal Status (Sham, Gdx) | 0.009129 | 0.9252 | 0.1140 | 0.7393 |
| Chromosomes (XX, XY) | **8.853** | **0.0100** | **18.92** | **0.0003** |
| *Two-way Interactions* |  |  |  |  |
| Gonadal Status*Chromosomes | 1.524 | 0.2374 | 0.02312 | 0.8807 |

*Summary of the two-way ANOVA results of main effects of sex chromosomes and gonadal status (Sham, Gdx), and their interactions on the expression of Kdm6a one-week post-HKSP immunization shown in Fig. 2F-G. Bold values indicate significance (p*<0.05).

**Table S5: Two-way ANOVA Fig. 3**

|  | ***Fig. 3C***  ***Xist*** | | ***Fig. 3D***  ***Uty*** | | ***Fig. 3E***  ***Sry*** | |
| --- | --- | --- | --- | --- | --- | --- |
| ***Effect*** | ***F*** | ***p value*** | ***F*** | ***p value*** | ***F*** | ***p value*** |
| *Main Effects* |  |  |  |  |  |  |
| Gonadal Sex (female, male) | 0.8098 | 0.3824 | **15.02** | **0.0015** | **82.70** | **<0.0001** |
| Chromosomes (XX, XY) | **2289** | **<0.0001** | **2037** | **<0.0001** | 1.411 | 0.2534 |
| *Two-way Interactions* |  |  |  |  |  |  |
| Gonadal Sex*Chromosomes | 0.9934 | 0.3347 | **15.02** | **0.0015** | 1.411 | 0.2534 |

*Summary of the two-way ANOVA results of main effects of gonadal sex and sex chromosomes and their interactions on gene expression of Xist, Uty, and Sry. Bold values indicate significance (p<0.05).*

**Table S6: Three-way ANOVA Fig. 3B-C**

|  | ***Fig. 3B CD138+*** | | ***Fig. 3C***  ***IgM Concentration*** | |
| --- | --- | --- | --- | --- |
| ***Effect*** | ***F*** | ***p value*** | ***F*** | ***p value*** |
| *Main Effects* |  |  |  |  |
| Gonadal Sex (female, male) | **186.5** | **<0.0001** | **4.232** | **0.0451** |
| Chromosomes (XX, XY) | 1.870 | 0.1782 | **40.13** | **<0.0001** |
| Treatment (DMSO, 2µM GSK J4, 2µM GSK J5) | **93.57** | **<0.0001** | **3.755** | **0.0305** |
| *Two-way Interactions* |  |  |  |  |
| Gonadal Sex*Chromosomes | **14.83** | **0.0004** | 2.111 | 0.1527 |
| Gonadal Sex*Treatment | **10.62** | **0.0002** | 0.9808 | 0.3824 |
| Chromosomes*Treatment | 0.1534 | 0.8582 | 0.8633 | 0.4282 |
| *Three-way Interactions* |  |  |  |  |
| Gonadal Sex*Chromosomes*Treatment | 1.979 | 0.1501 | 0.4872 | 0.6174 |

*Summary of the three-way ANOVA results of main effects of gonadal sex, sex chromosomes, and treatment, and their interactions on the frequencies of CD138+ plasma cells and IgM concentrations shown in Fig. 3B-C. Bold values indicate significance (p*<0.05).

**Table S7: Three-way ANOVA Fig. 5**

|  | ***CD138+*** | | | | ***IgM*** | | | |
| --- | --- | --- | --- | --- | --- | --- | --- | --- |
|  | ***Fig. 5A***  ***GSK J4*** | | ***Fig. 5C***  ***GSK J5*** | | ***Fig. 5B***  ***GSK J4*** | | ***Fig. 5D***  ***GSK J5*** | |
| ***Effect*** | ***F*** | ***p value*** | ***F*** | ***p value*** | ***F*** | ***p value*** | ***F*** | ***p value*** |
| *Main Effects* |  |  |  |  |  |  |  |  |
| Chromosomes (XX, XY) | 3.385 | 0.0697 | **4.943** | **0.0292** | **65.65** | **<0.0001** | **48.82** | **<0.0001** |
| Gonadal Sex (female, male) | **265.4** | **<0.0001** | **248.3** | **<0.0001** | 2.021 | 0.1600 | **4.811** | **0.0319** |
| Treatment (Naïve, DMSO,  GSK J4/GSK J5) | **254.5** | **<0.0001** | **270.4** | **<0.0001** | 1.245 | 0.3008 | 1.284 | 0.2874 |
| *Two-way Interactions* |  |  |  |  |  |  |  |  |
| Chromosomes*Gonadal Sex | **12.93** | **0.0006** | **17.27** | **<0.0001** | **5.478** | **0.0224** | 2.418 | 0.1249 |
| Chromosomes*Treatment | 0.8932 | 0.4724 | **3.333** | **0.0144** | 0.4009 | 0.7528 | 0.3786 | 0.7687 |
| Gonadal Sex*Treatment | **14.05** | **<0.0001** | **8.993** | **<0.0001** | 0.7826 | 0.5080 | 0.6337 | 0.5960 |
| *Three-way Interactions* |  |  |  |  |  |  |  |  |
| Chromosomes*Gonadal  Sex*Treatment | 2.008 | 0.1020 | 1.837 | 0.1306 | 0.0438 | 0.9877 | 0.3149 | 0.8145 |

*Summary of the three-way ANOVA results of main effects of sex chromosomes, gonadal sex, and treatment (Naïve, DMSO, and 0.25µM, 0.5 µM, or 2 µM GSK J4 or GSK J5), and their interactions on CD138+ plasma cells and IgM production shown in Fig. 5. Bold values indicate significance (p<0.05).*

**Table S8: Three-way ANOVA Fig. 4A**

| ***Effect*** | ***F*** | ***p value*** |
| --- | --- | --- |
| *Main Effects* |  |  |
| Gonadal Sex (female, male) | **119.6** | **<0.0001** |
| Chromosomes (XX, XY) | **6.451** | **0.0129** |
| Treatment (No Abx, Abx) | **19.95** | **<0.0001** |
| *Two-way Interactions* |  |  |
| Gonadal Sex*Chromosomes | **6.014** | **0.0162** |
| Gonadal Sex*Treatment | **13.95** | **0.0003** |
| Chromosomes*Treatment | 7.000 | 0.097 |
| *Three-way Interactions* |  |  |
| Gonadal Sex*Chromosomes*Treatment | 3.310 | 0.0723 |

*Summary of the three-way ANOVA results of main effects of gonadal sex, sex chromosomes, and antibiotics (Abx) treatment, and their interactions on the number of IgM-secreting B cells one-week post-HKSP immunization shown in Fig 4A. Bold values indicate significance (p<0.05).*

**Table S9: Two-way ANOVA Fig. 4B-C**

|  | ***Fig. 4B***  ***Females*** | | ***Fig. 4C***  ***Males*** | |
| --- | --- | --- | --- | --- |
| ***Effect*** | ***F*** | ***p value*** | ***F*** | ***p value*** |
| *Main Effects* |  |  |  |  |
| Chromosomes (XX, XY) | 3.889 | 0.0573 | 0.02638 | 0.8716 |
| Treatment (No Abx, Abx) | **10.50** | **0.0028** | **7.434** | **0.0086** |
| *Two-way Interactions* |  |  |  |  |
| Chromosomes*Treatment | 3.111 | 0.0873 | **9.361** | **0.0034** |

*Summary of the two-way ANOVA results of main effects sex chromosomes and antibiotics treatment when separated by sex, and their interactions on the number of IgM-secreting B cells one-week post-HKSP immunization shown in Fig 4B-C. Bold values indicate significance (p<0.05).*

**Table S10: Dunn’s multiple comparisons Fig. 5A-B**

| ***Comparison*** | ***OTUs*** | ***Shannon Diversity Index*** |
| --- | --- | --- |
| *Intact* |  |  |
| XXF vs. XYF | >0.999 | >0.999 |
| XXM vs. XYM | >0.999 | >0.999 |
| *Gonadectomized* |  |  |
| XXF vs. XYF | >0.999 | >0.999 |
| XXM vs. XYM | >0.999 | >0.999 |
| *XX* |  |  |
| F Intact vs. Gdx | **0.0145** | 0.1439 |
| M Intact vs. Gdx | >0.999 | >0.999 |
| *XY* |  |  |
| F Intact vs. Gdx | >0.999 | >0.999 |
| M Intact vs. Gdx | >0.999 | >0.999 |

*Summary of the Dunn’s multiple comparisons tests of gut microbiota alpha diversity shown in Fig. 5A-B. Bold values indicate significance (p<0.05). Gdx = gonadectomized.*

**Table S11: FCG microbiome diversity: Statistical analysis of Bray-Curtis distances in Intact vs. Gonadectomized**

| **Group 1** | **Group 2** | **Sample Size** | **Permutations** | **Pseudo-F** | **p-value** | **q-value** |
| --- | --- | --- | --- | --- | --- | --- |
| XXF Gdx | XXF Intact | 17 | 999 | 4.078913 | 0.001 | 0.0056 |
|  | XXM Gdx | 12 | 999 | 2.557224 | 0.006 | 0.012923 |
|  | XXM Intact | 18 | 999 | 3.447812 | 0.001 | 0.0056 |
|  | XYF Gdx | 10 | 999 | 2.555007 | 0.027 | 0.034364 |
|  | XYF Intact | 14 | 999 | 3.303385 | 0.005 | 0.012727 |
|  | XYM Gdx | 12 | 999 | 3.103468 | 0.008 | 0.014933 |
|  | XYM Intact | 16 | 999 | 2.656753 | 0.006 | 0.012923 |
| XXF Intact | XXM Gdx | 17 | 999 | 3.686444 | 0.001 | 0.0056 |
|  | XXM Intact | 23 | 999 | 2.300265 | 0.003 | 0.0105 |
|  | XYF Gdx | 15 | 999 | 3.422382 | 0.002 | 0.008 |
|  | XYF Intact | 19 | 999 | 1.763117 | 0.034 | 0.039667 |
|  | XYM Gdx | 17 | 999 | 1.691083 | 0.032 | 0.038957 |
|  | XYM Intact | 21 | 999 | 2.086596 | 0.005 | 0.012727 |
| XXM Gdx | XXM Intact | 18 | 999 | 1.754152 | 0.024 | 0.033333 |
|  | XYF Gdx | 10 | 999 | 1.878096 | 0.02 | 0.029474 |
|  | XYF Intact | 14 | 999 | 3.94989 | 0.001 | 0.0056 |
|  | XYM Gdx | 12 | 999 | 2.119504 | 0.014 | 0.021778 |
|  | XYM Intact | 16 | 999 | 1.510759 | 0.075 | 0.077778 |
| XXM Intact | XYF Gdx | 16 | 999 | 2.588798 | 0.005 | 0.012727 |
|  | XYF Intact | 20 | 999 | 3.606767 | 0.001 | 0.0056 |
|  | XYM Gdx | 18 | 999 | 2.007341 | 0.011 | 0.01925 |
|  | XYM Intact | 22 | 999 | 1.276936 | 0.19 | 0.19 |
| XYF Gdx | XYF Intact | 12 | 999 | 3.533556 | 0.002 | 0.008 |
|  | XYM Gdx | 10 | 999 | 3.034554 | 0.007 | 0.014 |
|  | XYM Intact | 14 | 999 | 1.973139 | 0.025 | 0.033333 |
| XYF Intact | XYM Gdx | 14 | 999 | 1.661752 | 0.055 | 0.0616 |
|  | XYM Intact | 18 | 999 | 2.115544 | 0.014 | 0.021778 |
| XYM Gdx | XYM Intact | 16 | 999 | 1.563419 | 0.073 | 0.077778 |

*Comparison of index distances using QIIME2 plugins using PERMANOVA supplemental to Table S3 for gonadally intact and gonadectomized (Gdx) animals.*

**Table S12: Relative Abundancies Fig. 5C**

|  | XXF (%) | XYF (%) | XXM (%) | XYM (%) |
| --- | --- | --- | --- | --- |
| Bacteriodaceae | 6.62 ± 3.13 | 4.98 ± 2.78 | 9.01 ± 6.79 | 9.30 ± 2.19 |
| Muribaculaceae | 49.48 ± 1.49 | 49.74 ± 10.53 | 39.36 ± 6.29 | 32.49 ± 6.94 |
| Provetellaceae | 2.288 ± 1.05 | 2.03 ± 1.70 | 2.25 ± 0.68 | 2.25 ± 0.80 |
| Rikennaceae | 7.40 ± 1.05 | 4.55 ± 1.83 | 8.62 ± 2.38 | 11.12 ± 5.98 |
| Tannerellaceae | 1.14 ± 1.22 | 0.61 ± 0.69 | 0.48 ± 0.36 | 0.99 ± 1.042 |
| Lactobacillaceae | 4.86 ± 2.73 | 3.65 ± 3.27 | 6.48 ± 3.30 | 10.54 ± 7.24 |
| Lachnospiraceae | 16.80 ± 4.65 | 21.32 ± 14.23 | 17.77 ± 9.71 | 20.03 ± 7.13 |
| Peptococcaceae | 0.06 ± 0.01 | 0.32 ± 0.30 | 0.64 ± 0.44 | 0.60 ± 049 |
| Ruminococcaceae | 5.01 ± 2.11 | 5.92 ± 1.61 | 4.49 ± 1.49 | 4.79 ± 2.95 |
| Erysipelotrichaceae | 1.78 ± 2.11 | 1.95 ± 1.47 | 2.22 ± 1.71 | 2.20 ± 1.1 |
| Burkholderiaceae | 0.45 ± 0.18 | 0.75 ± 0.56 | 0.83 ± 0.85 | 0.63 ± 0.35 |
| Tenericutes-uncultured | 0.84 ± 0.09 | 0.18 ± 0.10 | 1.08 ± 1.90 | 0.07 ± 0.7 |
| Bifidobacteriaceae | 0.00 ± 0.00 | 0.33 ± 0.58 | 1.72 ± 2.02 | 0.25 ± 0.24 |

*Percent abundancies ± standard deviation of the top 11 families identified in the gut microbiome by 16s rRNA sequencing in FCG mice as visualized in Fig. 5C.*

**Table S13: Two-way ANOVA Fig. 6**

|  | ***Acetate*** | | ***Butyrate*** | | ***Propionate*** | | ***2-Methylbutyric acid*** | |
| --- | --- | --- | --- | --- | --- | --- | --- | --- |
| ***Effect*** | ***F*** | ***p value*** | ***F*** | ***p value*** | ***F*** | ***p value*** | ***F*** | ***p value*** |
| *Main Effects* |  |  |  |  |  |  |  |  |
| Chromosomes (XX, XY) | 0.3483 | 0.5598 | 0.6157 | 0.4392 | 0.6516 | 0.4263 | 0.1562 | 0.6957 |
| Gonadal Sex (female, male) | **13.09** | **0.0012** | **11.29** | **0.0023** | 0.9972 | 0.3265 | **7.937** | **0.0088** |
| *Two-way Interactions* |  |  |  |  |  |  |  |  |
| Chromosomes*Gonadal Sex | 0.7414 | 0.3956 | 3.061 | 0.0911 | 0.0499 | 0.8249 | 1.088 | 0.3058 |

|  | ***Hexanoic acid*** | | ***Isobutyric acid*** | | ***Isovaleric acid*** | |
| --- | --- | --- | --- | --- | --- | --- |
| ***Effect*** | ***F*** | ***p value*** | ***F*** | ***p value*** | ***F*** | ***p value*** |
| *Main Effects* |  |  |  |  |  |  |
| Chromosomes (XX, XY) | 0.0614 | 0.8061 | 0.0225 | 0.8818 | 0.0048 | 0.9451 |
| Gonadal Sex (female, male) | **10.23** | **0.0034** | **13.19** | **0.0011** | **11.23** | **0.0023** |
| *Two-way Interactions* |  |  |  |  |  |  |
| Chromosomes*Gonadal Sex | 0.9617 | 0.3352 | 0.8638 | 0.3606 | 0.7675 | 0.3885 |

*Summary of the two-way ANOVA results of main effects of gonadal sex and sex chromosomes and their interactions on SCFA levels measured by LC-MS/MS shown in Fig. 6. Bold values indicate significance (p<0.05).*

**Table S14: Three-way ANOVA Fig. 7D-E**

|  | ***Fig. 7D***  ***Inulin vs. SCFA-producing bacteria*** | | ***Fig. 7E***  ***Inulin vs. Inulin + SCFA-producing bacteria*** | |
| --- | --- | --- | --- | --- |
| ***Effect*** | ***F*** | ***p value*** | ***F*** | ***p value*** |
| *Main Effects* |  |  |  |  |
| Gonadal Sex (female, male) | 0.9998 | 0.3262 | 2.179 | 0.1524 |
| Chromosomes (XX, XY) | 3.595 | 0.0687 | **22.60** | **<0.0001** |
| Treatment (Inulin,  SCFA-producing bacteria) | **10.87** | **0.0026** | **15.84** | **0.0005** |
| *Two-way Interactions* |  |  |  |  |
| Gonadal Sex*Chromosomes | 2.634 | 0.1162 | 2.561 | 0.1221 |
| Gonadal Sex*Treatment | 0.0011 | 0.9733 | 0.03786 | 0.8473 |
| Chromosomes*Treatment | 0.4003 | 0.5322 | **10.16** | **0.0038** |
| *Three-way Interactions* |  |  |  |  |
| Gonadal Sex*Chromosomes*  Treatment | 2.564 | 0.1210 | 2.648 | 0.1162 |

*Summary of the three-way ANOVA results of main effects of gonadal sex, sex chromosomes, and treatment with inulin and/or SCFA-producing bacteria, and their interactions on the number of IgM-secreting B cells shown in Fig 7. Bold values indicate significance (p<0.05).*

**Table S15: Three-way ANOVA Fig. 7F-H and Fig. 7**

|  | ***Acetate*** | | ***Butyrate*** | | ***Propionate*** | | ***2-Methylbutyric acid*** | |
| --- | --- | --- | --- | --- | --- | --- | --- | --- |
| ***Effect*** | ***F*** | ***p value*** | ***F*** | ***p value*** | ***F*** | ***p value*** | ***F*** | ***p value*** |
| *Main Effects* |  |  |  |  |  |  |  |  |
| Chromosomes (XX, XY) | 3.616 | 0.0717 | 1.043 | 0.3194 | **9.861** | **0.0052** | 2.102 | 0.1627 |
| Gonadal Sex (female, male) | 0.6605 | 0.4260 | 0.0006 | 0.9812 | 0.1226 | 0.7299 | 0.0515 | 0.8228 |
| Treatment (No reconstitution,  + reconstitution) | **62.21** | **<0.0001** | **20.05** | **0.0002** | **28.03** | **<0.0001** | **17.44** | **0.0005** |
| *Two-way Interactions* |  |  |  |  |  |  |  |  |
| Chromosomes*Gonadal Sex | 0.0113 | 0.9166 | 0.3694 | 0.5502 | 0.3824 | 0.5433 | 1.397 | 0.2511 |
| Chromosomes*Treatment | 3.743 | 0.0673 | 1.057 | 0.3162 | **10.08** | **0.0048** | 1.622 | 0.2174 |
| Gonadal Sex*Treatment | 0.5976 | 0.4485 | 0.0001 | 0.9757 | 0.1481 | 0.7044 | 0.0624 | 0.8053 |
| *Three-way Interactions* |  |  |  |  |  |  |  |  |
| Chromosomes*Gonadal Sex*  Treatment | 0.0187 | 0.8925 | 0.3610 | 0.5547 | 0.3406 | 0.5660 | 1.334 | 0.2616 |

|  | ***Hexanoic acid*** | | ***Isobutyric acid*** | | ***Isovaleric acid*** | |
| --- | --- | --- | --- | --- | --- | --- |
| ***Effect*** | ***F*** | ***p value*** | ***F*** | ***p value*** | ***F*** | ***p value*** |
| *Main Effects* |  |  |  |  |  |  |
| Chromosomes (XX, XY) | 0.0403 | 0.8429 | 1.100 | 0.3067 | 0.1165 | 0.7364 |
| Gonadal Sex (female, male) | 0.3601 | 0.5552 | 0.1291 | 0.7232 | 0.6269 | 0.4378 |
| Treatment (No reconstitution,  + reconstitution) | 3.707 | 0.0685 | **14.58** | **0.0011** | **11.05** | **0.034** |
| *Two-way Interactions* |  |  |  |  |  |  |
| Chromosomes*Gonadal Sex | 0.7069 | 0.4104 | 0.7543 | 0.3954 | 1.053 | 0.3171 |
| Chromosomes*Treatment | 0.3710 | 0.5493 | 1.041 | 0.3198 | 0.1711 | 0.6835 |
| Gonadal Sex*Treatment | 0.6275 | 0.4376 | 0.1187 | 0.7340 | 0.6734 | 0.4215 |
| *Three-way Interactions* |  |  |  |  |  |  |
| Chromosomes*Gonadal Sex*  Treatment | 0.6108 | 0.4436 | 0.7801 | 0.3876 | 0.9947 | 0.3305 |

*Summary of the three-way ANOVA results of main effects of gonadal sex, sex chromosomes, and treatment, and their interactions on SCFA levels measured by LC-MS/MS shown in Fig. 7F-H and Fig. 7. Bold values indicate significance (p<0.05).*

**Table S16: Three-way ANOVA Fig. 8**

|  | ***Fig. 8A***  ***% Living Cells*** | | ***Fig. 8B***  ***# CD138+*** | | ***Fig. 8C***  ***IgM*** | |
| --- | --- | --- | --- | --- | --- | --- |
| ***Effect*** | ***F*** | ***p value*** | ***F*** | ***p value*** | ***F*** | ***p value*** |
| *Main Effects* |  |  |  |  |  |  |
| Gonadal Sex (female, male) | **260.4** | **<0.0001** | 2.430 | 0.1256 | **39.96** | **<0.0001** |
| Chromosomes (XX, XY) | 3.848 | 0.0556 | 0.3350 | 0.5654 | **26.79** | **<0.0001** |
| Treatment (+/- propionate) | **43.31** | **<0.0001** | **79.96** | **<0.0001** | **65.29** | **<0.0001** |
| *Two-way Interactions* |  |  |  |  |  |  |
| Gonadal Sex*Chromosomes | **6.222** | **0.0161** | 0.06464 | 0.8004 | 1.820 | 0.1836 |
| Gonadal Sex*Treatment | **12.86** | **<0.0001** | 1.269 | 0.2957 | 0.6368 | 0.5950 |
| Chromosomes*Treatment | 0.1390 | 0.9362 | 0.7893 | 0.5058 | 1.957 | 0.1330 |
| *Three-way Interactions* |  |  |  |  |  |  |
| Gonadal Sex*Chromosomes*Treatment | 0.06549 | 0.9779 | 0.05622 | 0.9823 | 0.3440 | 0.7936 |

*Summary of the three-way ANOVA results of main effects of gonadal sex, sex chromosomes, and treatment with propionate, and their interactions on cell viability, CD138+ plasma cell numbers, and IgM production shown in Fig 8. Bold values indicate significance (p<0.05).*

**Table S17: Three-way ANOVA Fig. 8**

|  | ***% Living Cells*** | | ***# CD138+*** | | ***IgM*** | |
| --- | --- | --- | --- | --- | --- | --- |
| ***Effect*** | ***F*** | ***p value*** | ***F*** | ***p value*** | ***F*** | ***p value*** |
| *Main Effects* |  |  |  |  |  |  |
| Chromosomes (XX, XY) | 3.848 | 0.0556 | 0.3350 | 0.5654 | **26.79** | **<0.0001** |
| Gonadal Sex (female, male) | **260.4** | **<0.0001** | 2.430 | 0.1256 | **39.96** | **<0.0001** |
| Treatment (-/+ 0.5mM, 1mM,  or 2mM propionate) | **43.31** | **<0.0001** | **79.96** | **<0.0001** | **65.29** | **<0.0001** |
| *Two-way Interactions* |  |  |  |  |  |  |
| Chromosomes*Gonadal Sex | **6.222** | **0.0161** | 0.06464 | 0.8004 | 1.820 | 0.1836 |
| Chromosomes*Treatment | 0.1390 | 0.9362 | 0.7893 | 0.5058 | 1.957 | 0.1330 |
| Gonadal Sex*Treatment | **12.86** | **<0.0001** | 1.269 | 0.2957 | 0.6368 | 0.5950 |
| *Three-way Interactions* |  |  |  |  |  |  |
| Chromosomes*Gonadal Sex*  Treatment | 0.06549 | 0.9779 | 0.05622 | 0.9823 | 0.3440 | 0.7936 |

*Summary of the three-way ANOVA results of main effects of gonadal sex, sex chromosomes, and propionate treatment, and their interactions shown in Fig. 8. Bold values indicate significance (p<0.05).*
